# Hazelnut Husk Waste-Derived Activated Carbon for Adsorption of a Remdesivir-Containing Commercial Formulation: Equilibrium, Kinetic, and Thermodynamic Studies

**DOI:** 10.3390/molecules31213801

**Published:** 2026-10-22

**Authors:** Serdar Güngör, İbrahim Bulduk

**Affiliations:** 1 Department of Medical Microbiology, Faculty of Medicine, Bilecik Şeyh Edebali University, Bilecik 11100, Turkey; 2 Department of Chemical Engineering, Faculty of Engineering, Afyon Kocatepe University, Afyonkarahisar 03000, Turkey; ibrahim.bulduk@aku.edu.tr

**Keywords:** remdesivir, activated carbon, hazelnut husk, biomass valorization, agro-waste, adsorption mechanism, emerging contaminants, water remediation

## Abstract

The occurrence of antiviral pharmaceutical residues in aquatic environments has raised increasing concern regarding their environmental persistence and removal by conventional wastewater treatment processes. In this study, activated carbon was prepared from hazelnut husk agro-waste using KOH-assisted chemical activation and evaluated for the adsorption of a remdesivir-containing commercial pharmaceutical formulation from aqueous media. Textural and morphological characterization by Brunauer–Emmett–Teller (BET), scanning electron microscopy (SEM), and energy-dispersive X-ray spectroscopy (EDX) indicated the development of a porous carbon structure with a BET surface area of 441.72 ± 7.72 m^2^ g^−1^. Batch adsorption experiments were performed to investigate the effects of adsorbent dose, solution pH, initial concentration, contact time, and temperature. The equilibrium data were described by Langmuir, Temkin, Freundlich, and Dubinin–Radushkevich models using nonlinear regression and multiple error functions. The Langmuir model provided the best statistical description among the tested models, with a calculated maximum capacity of 199.11 mg g^−1^ (R^2^ = 0.9759), whereas the highest experimentally determined uptake reported in the equilibrium experiments was 205.60 mg g^−1^. The adsorption kinetics were best described by the pseudo-second-order model (R^2^ = 0.999); however, this empirical kinetic fit was not interpreted as independent proof of possible chemical contribution. Thermodynamic analysis based on the temperature-dependent equilibrium constants yielded a negative enthalpy change of −49.29 kJ mol^−1^ and negative Gibbs free-energy values over the investigated temperature range, indicating a favorable and exothermic adsorption process under the experimental conditions. The results suggest that the adsorption behavior may involve a combination of pore accessibility and non-covalent adsorbent–adsorbate interactions. Because a commercial formulation containing sulfobutylether-β-cyclodextrin (SBECD) was used, possible contributions of the excipient to adsorption and chromatographic response cannot be completely excluded. Therefore, the present findings should be regarded as an initial assessment of the adsorption behavior of a remdesivir-containing pharmaceutical formulation rather than direct evidence of treatment performance in real wastewater.

## 1. Introduction

Sustainable management of water resources has become one of the most important environmental problems of our time. Population growth, urbanization, industrialization, and the expansion of healthcare services significantly increase the anthropogenic pollutant load on water resources. In recent years, pharmaceutical active compounds (PACs) have become one of the most commonly detected emerging pollutants in environmental settings [1,2]. A significant portion of the drugs used in human and veterinary medicine are excreted without being metabolized or partially metabolized, reaching sewage systems and not being fully removed by conventional wastewater treatment plants [3]. Therefore, pharmaceutical residues are detected in surface waters, groundwater, sediments, and even drinking water sources [4]. The continuous presence of pharmaceutical pollutants in the environment leads to significant environmental risks such as bioaccumulation, ecotoxicity, endocrine-disrupting effects, and the development of microbial resistance [5,6].

Following the COVID-19 pandemic, the use of antiviral drugs has increased significantly on a global scale [7]. Among these drugs, Remdesivir (RDV) is a nucleotide analog that acts as an RNA-dependent RNA polymerase inhibitor and has been widely used in the treatment of COVID-19 [8]. Increased consumption during the pandemic has increased the likelihood of Remdesivir and its metabolites reaching wastewater systems [9]. Remdesivir is considered a significant pollutant candidate in adsorption processes due to its low solubility in water (log P ≈ 3.2), molecular weight of 602.6 g/mol, and multiple ionizable groups (pKa ≈ 3.3) [8,9]. The environmental fate of antiviral drugs, the toxicity of their metabolic products, and the potential contributions of these compounds to the development of viral resistance in aquatic ecosystems are among the growing concerns, although this drug group has been studied less compared to other pharmaceutical classes [10]. Recent studies have shown that a significant portion of antiviral drugs cannot be completely removed by conventional biological treatment systems and can persist in environmental settings [11]. Therefore, antiviral drugs are considered among the new generation of environmental pollutants that need to be investigated as a priority today [12].

Various technologies such as membrane filtration, advanced oxidation processes, electrochemical methods, and adsorption are used for the removal of pharmaceutical pollutants [13]. Among these methods, adsorption is considered one of the most promising methods due to its high removal efficiency, low energy requirement, ease of application, and effectiveness against a wide range of pollutants [14]. Activated carbons are widely used in the removal of pharmaceutical pollutants thanks to their high surface areas, improved pore structures, and surface functional groups [15]. The high cost of commercial activated carbons necessitates the development of cheaper and more sustainable alternatives to these materials. In this context, the evaluation of agricultural wastes as activated carbon precursor materials offers a solution to the waste management problem and enables the production of low-cost adsorbents [16]. In this context, transforming agricultural and forestry residues into functional porous carbon materials represents an ideal ‘waste-to-resource’ strategy that aligns seamlessly with green chemistry and circular bioeconomy principles [16,17]. Biomass precursors inherently possess carbon-rich, lignocellulosic frameworks that can be selectively engineered—via thermochemical and chemical activation routes—into hierarchical pore architectures with tailored surface heterogeneities [17,18]. Recent advances have highlighted that the distinct physicochemical merits of biomass-derived carbons, such as tunable micro/mesoporosity, surface oxygen functionalities, and high aromaticity, play a paramount role in governing mass transport dynamics and specific interfacial interactions during the uptake of complex emerging organic contaminants [17,18]. Consequently, rationally designed bio-based carbons have demonstrated extraordinary adsorptive performance, broad pH resilience, and high structural stability, serving as versatile, scalable, and economically feasible alternatives to fossil-derived commercial sorbents in advanced water decontamination platforms [15,18].

In recent years, it has been reported that activated carbons produced from palm kernels, walnut shells, grape seeds, rice hulls, and various agricultural wastes have shown high performance in drug removal [19,20,21,22]. For example, while adsorption capacities in the range of 150–250 mg/g in antibiotic removal were reported for activated carbons produced from walnut shells [20], removal efficiencies in the range of 100–180 mg/g in antiviral drugs were obtained with palm kernel-based activated carbons [19]. However, these studies reveal that the unique physicochemical properties of each biomass type directly affect the adsorption performance and that detailed mechanism studies are necessary for each pollutant-adsorbent pair. Among agricultural wastes, hazelnut husk is considered a very suitable precursor material for activated carbon production due to its lignocellulosic structure, high carbon content (~45–50%) and abundant availability [23]. Turkey is the leading producer of hazelnuts in the world with approximately 70% share, producing an average of 600,000–700,000 tons of hazelnuts annually. This production volume results in approximately 150,000–200,000 tons of hazelnut husk waste being generated annually, and the utilization of this waste is of great importance both economically and environmentally [23]. Although the removal of various organic pollutants with activated carbons produced from hazelnut shells or husks has been studied in the literature [24,25], there are a limited number of studies on the adsorption performance of these materials, especially on antiviral drugs. Among the chemical activation methods in activated carbon production, potassium hydroxide (KOH) is known as one of the most effective activators in obtaining high surface area and microporous carbon structures [24]. KOH activation promotes expansion and new pore formation in the carbon matrix with the formation of metallic potassium (K) and potassium carbonate (K_2_CO_3_) during carbonization, while simultaneously contributing to the development of micro- and mesoporosity with the release of H_2_ and CO gas [26,27]. The activated carbons obtained by this method can typically reach BET surface area values of 400–1500 m^2^/g, and this large surface area allows for high-capacity removal of pharmaceutical pollutants [28].

Although the removal of many pharmaceutical pollutants with activated carbons produced from various biomass materials has been studied in the literature, a study that comprehensively investigated the adsorption of Remdesivir using activated carbon produced from hazelnut husk by KOH activation from an isotherm, kinetic and thermodynamic perspective has not been found. Especially after the COVID-19 pandemic, the increase in the consumption of antiviral drugs and the growing concerns about the environmental fate of these compounds make it necessary to remove an important antiviral compound such as Remdesivir from aqueous environments with effective and sustainable methods. The fact that hazelnut husk is a low-cost, abundant and renewable waste material makes this study attractive both economically and environmentally.

The main objective of this study is to thoroughly evaluate the adsorption performance of activated carbon produced from hazelnut husks via KOH chemical activation in the removal of Remdesivir from aqueous solutions. In this context, the physicochemical properties of the produced activated carbon were characterized using BET surface area analysis, SEM, and EDX techniques. To elucidate the mechanism of the adsorption process, the effects of different adsorption parameters (adsorbent dose, pH, initial concentration, contact time, and temperature) were investigated; equilibrium data were evaluated using Langmuir, Freundlich, Temkin, and Dubinin–Radushkevich isotherm models, while kinetic data were evaluated using pseudo-first-order, pseudo-second-order, Elovich, and intraparticle diffusion models. Furthermore, the thermodynamic parameters (ΔG^0^, ΔH^0^, and ΔS^0^) of the adsorption process were calculated to investigate the nature of the process and its dependence on temperature. The findings aim to demonstrate the potential of hazelnut husk-based activated carbon as a sustainable, environmentally friendly, and high-performance adsorbent for removing antiviral pharmaceuticals such as Remdesivir from aqueous environments.

## 2. Results

### 2.1. Characterization Tests

#### 2.1.1. Textural and Porosity Characteristics

**Figure 1:**
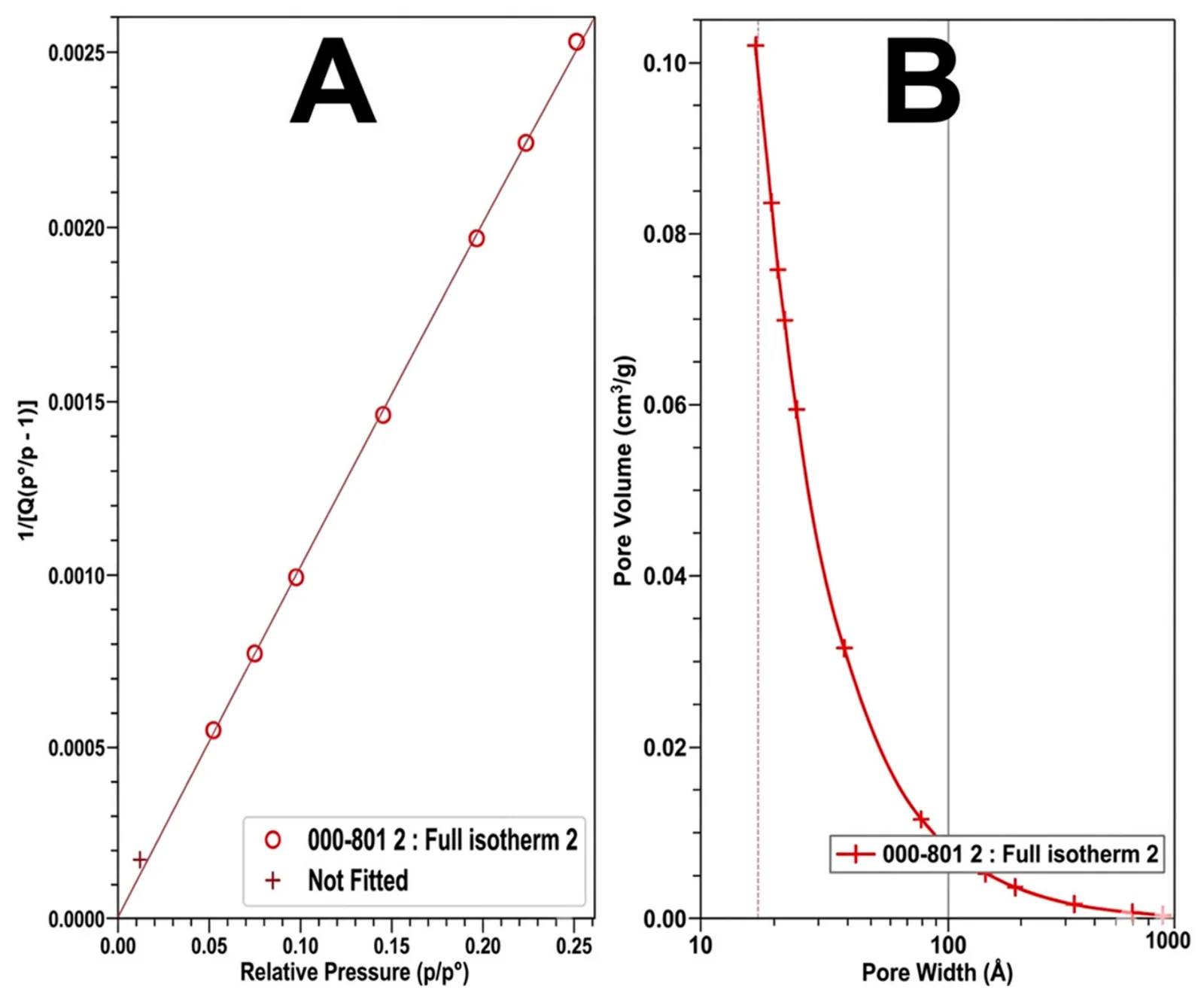
(**A**): BET surface area plot of the activated carbon sample (Full isotherm 2). The linear fit (R^2^ = 0.9993) is shown for the relative pressure range of 0.05–0.25 p/p° (**B**): BJH adsorption pore size distribution (dV/dlog(w) pore volume) of the activated carbon.

The porous architecture and textural properties of the hazelnut husk-derived activated carbon were evaluated from N_2_ adsorption–desorption measurements at 77 K. The material exhibited a BET specific surface area of 441.72 ± 7.72 m^2^ g^−1^, with a single-point surface area of 432.44 m^2^ g^−1^. The t-plot analysis yielded a micropore area of 142.17 m^2^ g^−1^ and a t-plot external surface area of 299.54 m^2^ g^−1^. Thus, the t-plot external surface area accounted for approximately 68% of the BET surface area. This value should be interpreted as the external surface contribution estimated by the t-plot method rather than as a direct measurement of geometrically accessible mesopore surface area. The single-point total pore volume was 0.2490 cm^3^ g^−1^ at p/p_0_ = 0.9946, while the micropore volume was 0.0658 cm^3^ g^−1^. The cumulative meso-/macropore volume obtained from the BJH adsorption analysis was 0.1020 cm^3^ g^−1^. The BET plot showed a high linearity (R^2^ = 0.9993), supporting the reproducibility of the BET fitting within the selected relative-pressure range (Figure 1A).

#### 2.1.2. Pore Volume Characteristics

The total pore volume of activated carbon was determined as 0.249 cm^3^/g using the single-point adsorption method at 0.9946 p/p°. T-graph analysis showed that micropore volume constituted a significant portion of this total, reaching 0.066 cm^3^/g. The cumulative volume attributed to meso- and macropores, calculated using the BJH adsorption method, is 0.102 cm^3^/g. These findings indicate that the total pore volume originates from both micropores and larger pores (meso/macropores) in approximately equal proportions.

#### 2.1.3. Pore Size Distribution

The average pore diameter calculated from the BET method was 22.54 Å for both the adsorption and desorption branches. The BJH analysis yielded average pore widths of 27.14 Å for the adsorption branch and 40.75 Å for the desorption branch. The BJH adsorption cumulative data indicated pore diameters ranging from approximately 17 to 3000 Å. The cumulative pore volume obtained from the adsorption branch (0.102 cm^3^ g^−1^) was higher than that obtained from the desorption branch (0.073 cm^3^ g^−1^), and the limited number of pore-spacing points obtained from the desorption branch suggests that the pore-size distribution should be interpreted cautiously.

The t-plot analysis independently indicated the presence of microporosity, with a micropore area of 142.17 m^2^ g^−1^ and a micropore volume of 0.0658 cm^3^ g^−1^. The combined BET, t-plot, and BJH results therefore indicate the coexistence of microporous and larger-pore domains in the activated carbon. Because DFT/NLDFT pore-size analysis was not performed in the present study, no DFT-based pore-size distribution is inferred. The pore-size interpretation is consequently restricted to the experimental information obtained from the BET, t-plot, and BJH analyses (Figure 1B).

#### 2.1.4. Isotherm Evaluation

**Figure 2:**
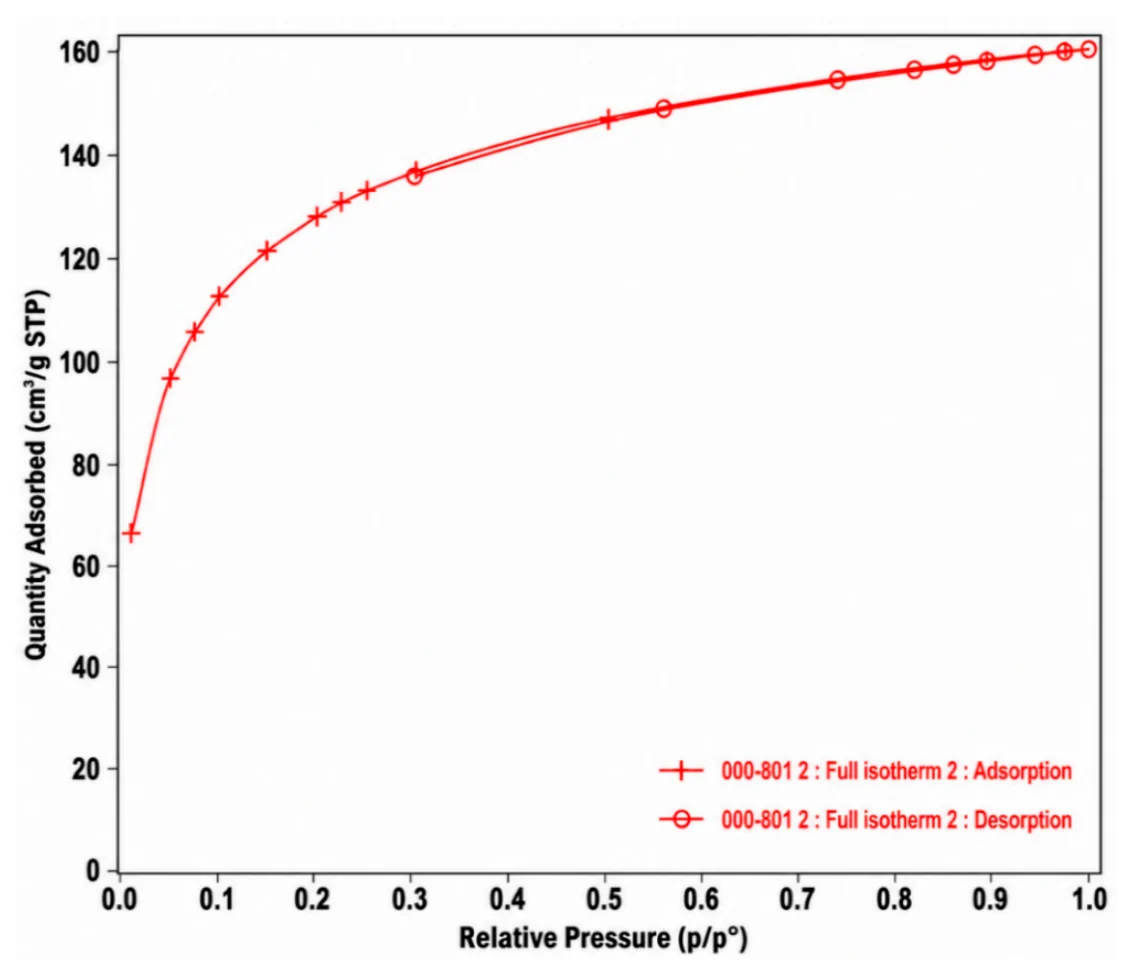
N_2_ adsorption–desorption isotherm of the activated carbon at 77 K. Closed symbols: adsorption; open symbols: desorption.

The obtained N_2_ adsorption–desorption isotherm exhibits the characteristic features of a Type IV (a) isotherm according to the IUPAC classification (Figure 2). A rapid increase in adsorption at low relative pressures (p/p° < 0.1) indicates the presence of micropores, while continuous adsorption at medium and high pressures (0.2–0.9 p/p°) supports the presence of mesopores. The isotherm exhibits an H4 type hysteresis loop. The H4 hysteresis loop is generally associated with narrow slit-shaped pores or micro-mesoporous materials. The lower slope of the desorption branch compared to the adsorption branch suggests possible pore-opening constrictions (ink bottle effect) or swelling events.

#### 2.1.5. Morphological Characteristics

**Figure 3:**
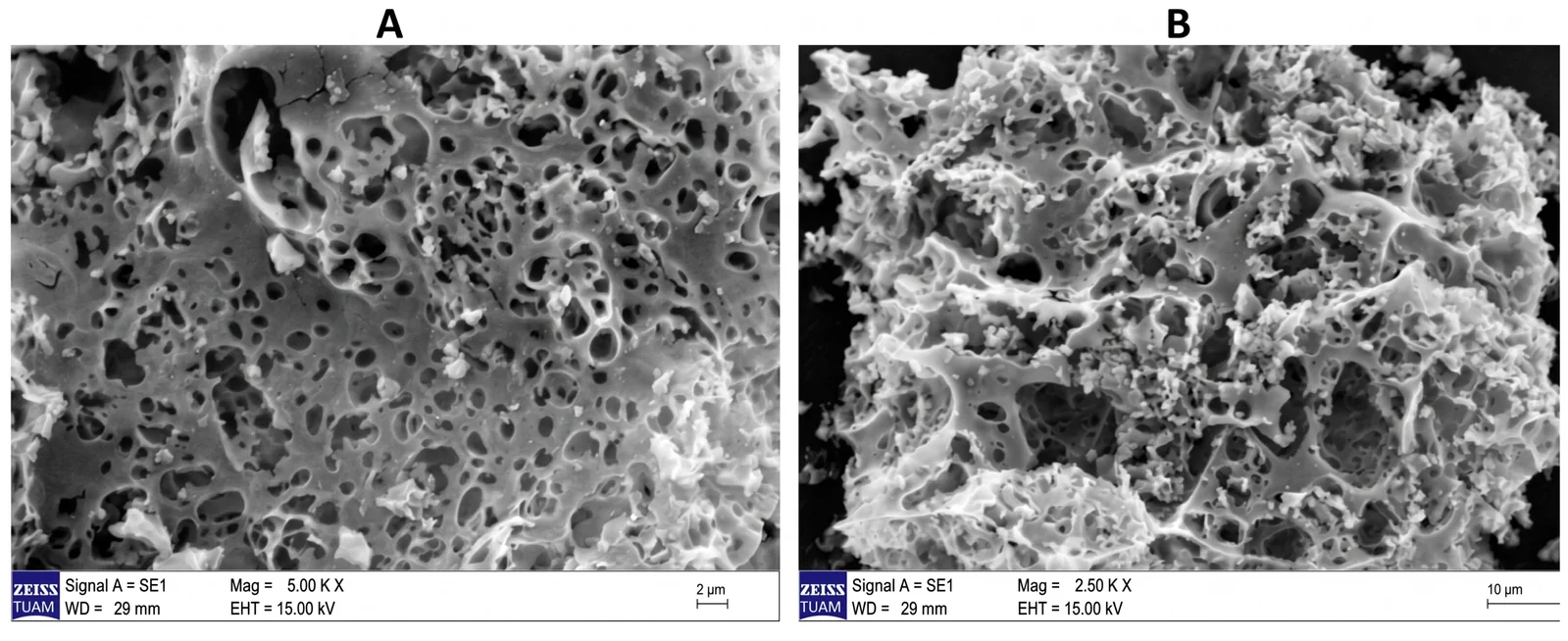
SEM images of the activated carbon at different magnifications: (**A**) low magnification, (**B**) medium magnification showing surface porosity.

SEM images taken at low, medium, and high magnifications revealed that the activated carbon particles have an irregular, angular, and blocky morphology (Figure 3). The particle surfaces exhibited a porous structure containing voids and cavities of various sizes. High-magnification images showed that some of these voids were irregularly distributed micro- and meso-sized pores, giving the material a spongy and rough texture. This morphology is consistent with the formation of voids resulting from the removal of volatile substances from the organic precursor (hazelnut husk) during the activation process.

#### 2.1.6. Elemental Composition (EDX)

**Figure 4:**
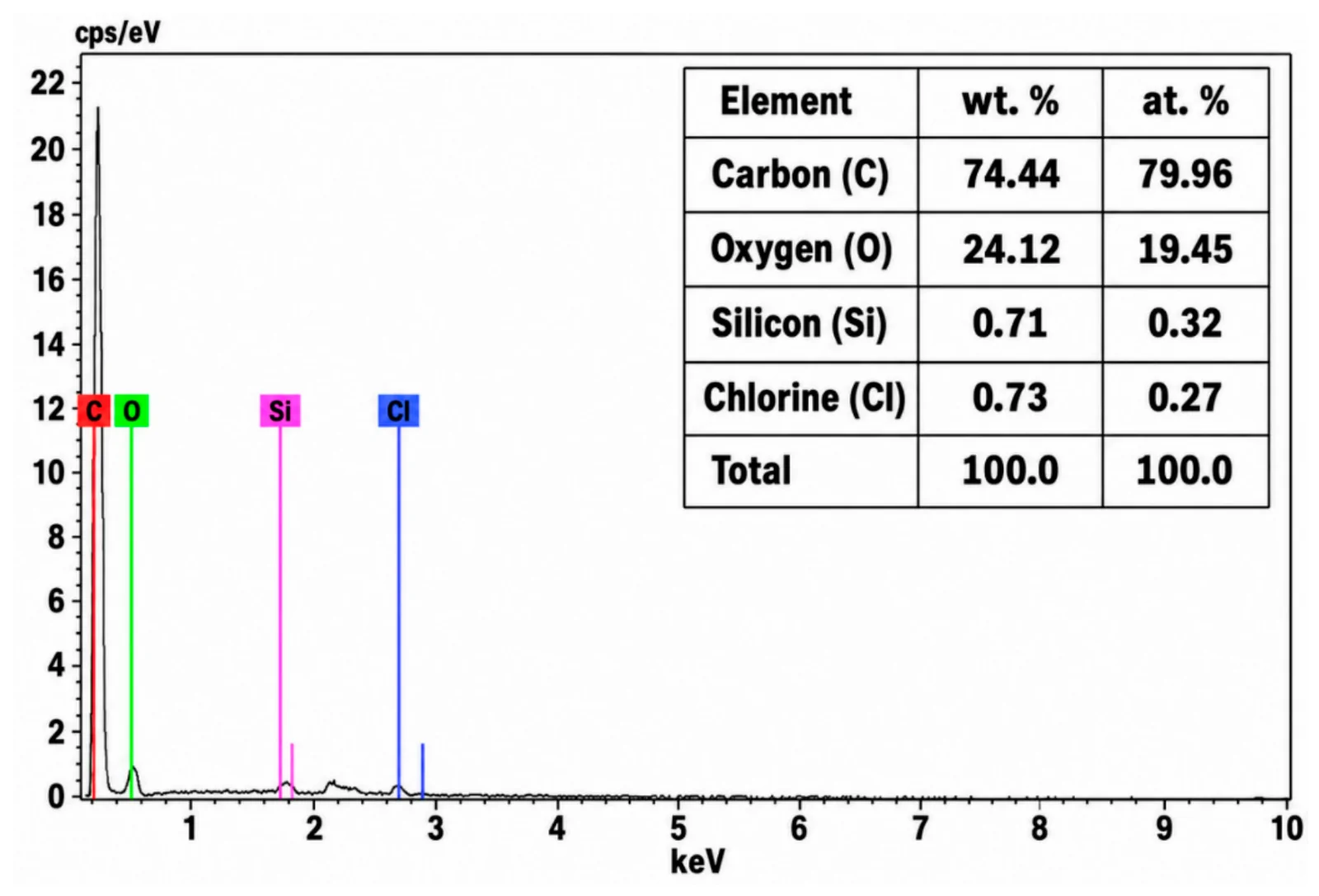
EDX spectrum of the activated carbon showing the elemental composition.

EDX analysis (Figure 4) showed that the activated carbon was predominantly composed of carbon (74.44 wt.%) and oxygen (24.12 wt.%), with minor amounts of silicon (0.71 wt.%) and chlorine (0.73 wt.%). The relatively high oxygen content indicates that oxygen-containing elemental species are present on the carbon surface. However, EDX provides elemental composition rather than the chemical-state information required to assign specific functional groups. Therefore, the presence of carboxyl, hydroxyl, carbonyl, or other oxygen-containing functionalities cannot be established solely from the EDX results. Such assignments would require complementary spectroscopic characterization, such as FTIR or XPS. The present EDX results are therefore interpreted only as evidence for the elemental presence of carbon, oxygen, silicon, and chlorine in the analyzed surface regions.

#### 2.1.7. Determination of pHpzc Value

**Figure 5:**
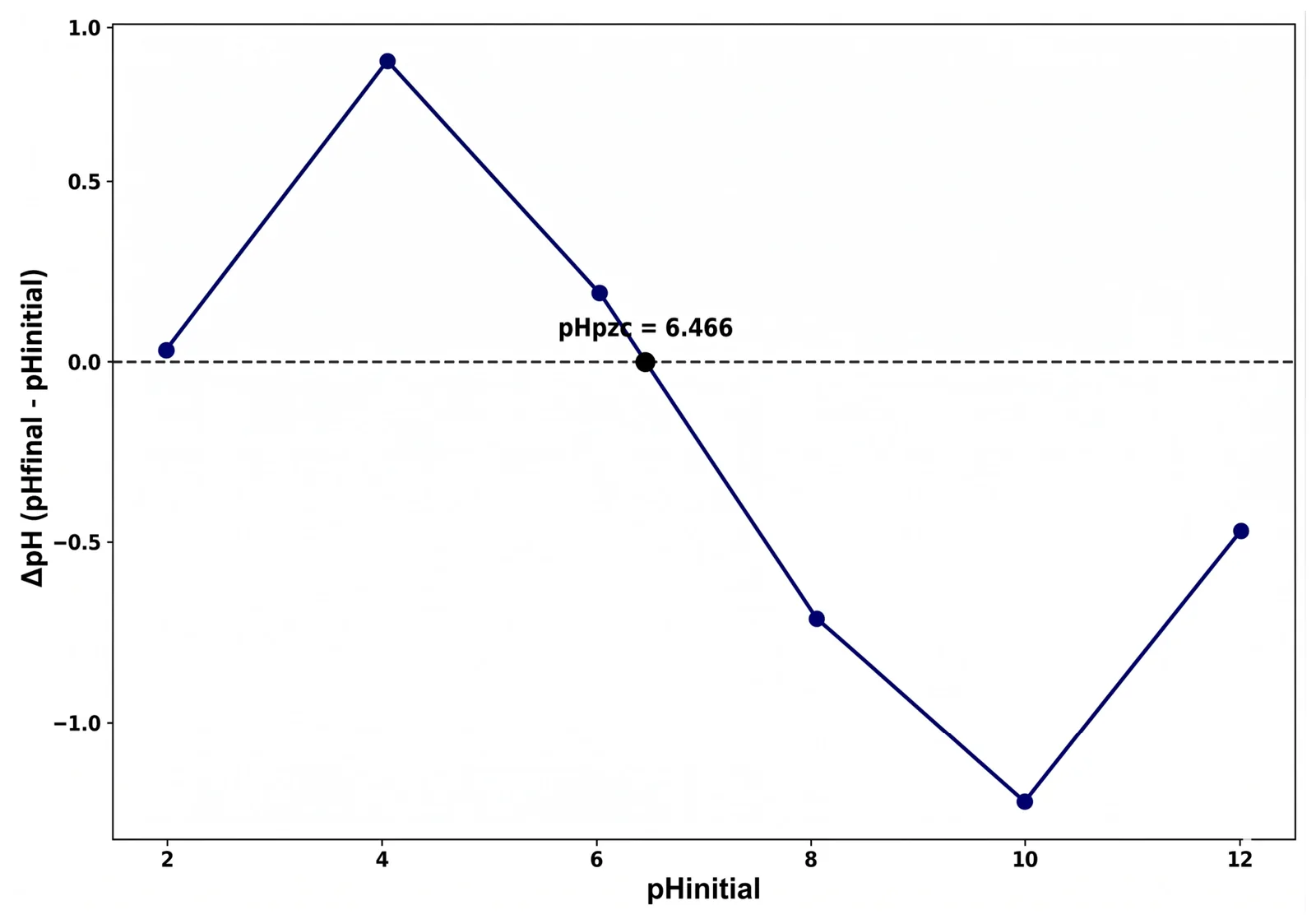
pH pZc graph of activated carbon.

The point of zero charge (pHpzc) of the hazelnut husk-derived activated carbon was determined to be 6.466 (Figure 5). Accordingly, the net surface charge is expected to be predominantly positive below this pH and predominantly negative above this pH under the experimental conditions used for the pHpzc determination.

The pHpzc value is relevant to interpreting the possible electrostatic contribution to adsorption; however, it should not be considered sufficient to define the complete surface chemistry or the molecular adsorption mechanism. In addition, the speciation of Remdesivir and the charge state of the adsorbent may vary with solution pH. Consequently, changes in electrostatic attraction or repulsion may occur across the investigated pH range. The relatively narrow variation in the experimentally measured adsorption capacity suggests that electrostatic interactions alone may not control the overall adsorption behavior. Other contributions, including pore accessibility and non-electrostatic interactions, may also participate.

Because the present study did not include XPS or other surface-sensitive spectroscopic characterization, the specific oxygen-containing functional groups responsible for adsorption cannot be identified unambiguously. The pHpzc and EDX results are therefore considered complementary evidence for interpreting the surface characteristics rather than direct proof of a particular molecular interaction.

### 2.2. Determination of Optimum Adsorption Conditions

Batch adsorption experiments were conducted to evaluate the performance of activated carbon produced from hazelnut husks in the adsorption of remdesivir from aqueous solutions. In this context, five basic parameters affecting adsorption efficiency were systematically investigated: adsorbent dose, solution pH, initial remdesivir concentration, contact time, and temperature.

**Figure 6:**
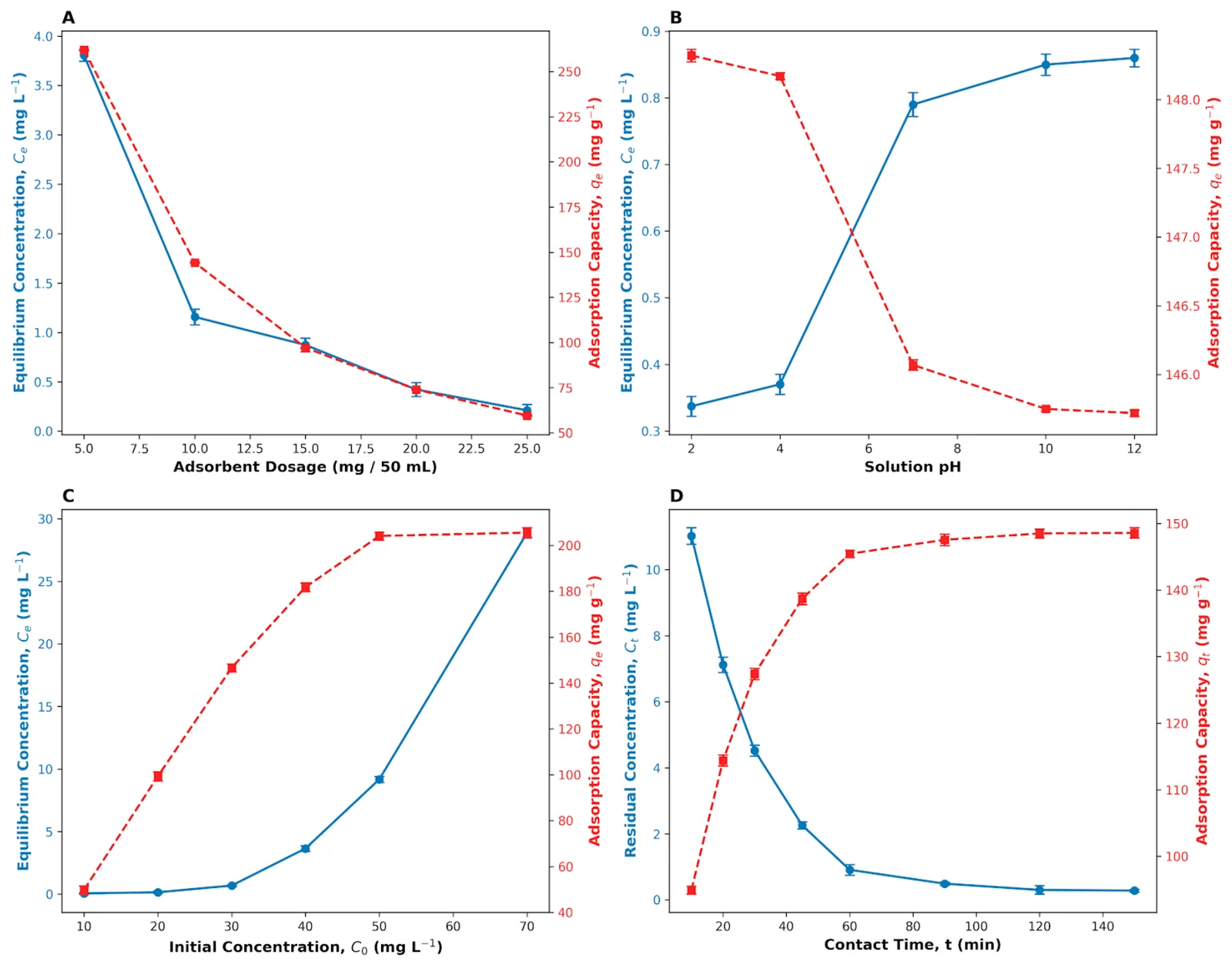
Effects of experimental variables on the adsorption behavior of the remdesivir-containing commercial formulation: (**A**) adsorbent dose, (**B**) solution pH, (**C**) initial formulation concentration, and (**D**) contact time. The adsorption capacity (q_e_, mg g^−1^) and equilibrium concentration (C_e_, mg L^−1^) are plotted on separate and explicitly labeled axes. All values are calculated from the corresponding experimental C_0_, C_e_, solution volume, and adsorbent mass using the adsorption-capacity equation described in Section 4.7.

The effect of adsorbent dose on Remdesivir removal was investigated by varying the amount of activated carbon from 5 to 25 mg per 50 mL of Remdesivir solution while maintaining an initial Remdesivir concentration of 30 mg L^−1^. The equilibrium concentration (C_e_) and adsorption capacity (q_e_) were calculated for each adsorbent dose using the mass-balance equation q_e_ = (C_0_ − C_e_) V/m. As shown in Figure 6A, increasing the adsorbent dose resulted in a progressive decrease in the residual Remdesivir concentration. The C_e_ value decreased from 3.807 mg L^−1^ at 5 mg adsorbent to 1.157, 0.873, 0.422, and 0.211 mg L^−1^ at adsorbent doses of 10, 15, 20, and 25 mg, respectively. Correspondingly, the removal efficiency increased from 87.31% at 5 mg to 96.14%, 97.09%, 98.59%, and 99.30% at 10, 15, 20, and 25 mg, respectively. In contrast to the removal efficiency, the equilibrium adsorption capacity expressed per unit mass of adsorbent decreased with increasing adsorbent dose. The calculated q_e_ decreased from 261.93 mg g^−1^ at 5 mg adsorbent to 144.22, 97.09, 73.95, and 59.58 mg g^−1^ at 10, 15, 20, and 25 mg, respectively. The corrected values are mathematically consistent with the initial concentration, equilibrium concentration, solution volume, and adsorbent mass used in the experiments. The simultaneous increase in removal efficiency and decrease in q_e_ with increasing adsorbent dose indicates that a larger amount of activated carbon provides a greater number of available adsorption sites and consequently promotes the removal of Remdesivir from the aqueous phase. However, because the total adsorbed amount is distributed over an increasing mass of adsorbent, the adsorption capacity calculated per gram of adsorbent decreases. Although the highest removal efficiency was obtained at 25 mg/50 mL (99.30%), the increase in removal efficiency beyond 10 mg/50 mL was relatively limited compared with the additional adsorbent requirement. Therefore, 10 mg/50 mL was considered a practical optimum dose, providing a high Remdesivir removal efficiency of 96.14% while avoiding unnecessary adsorbent consumption.

Solution pH (Figure 6B): The experimentally determined adsorption capacity varied only within a relatively narrow range, from 148.32 to 145.72 mg g^−1^, over the investigated pH range of 2–12. Although the overall change in adsorption capacity was limited, this observation does not imply that electrostatic interactions were identical throughout the entire pH range. The activated carbon exhibited a pHpzc of 6.466, indicating a change in the predominant surface charge state around this pH. At the same time, Remdesivir-containing species may undergo changes in protonation state as the solution pH changes. Consequently, electrostatic attraction and repulsion may contribute differently at different pH values.

The relatively stable adsorption capacity suggests that these electrostatic effects were not the sole controlling factor under the experimental conditions. Pore accessibility and non-electrostatic interactions, such as dispersion, π-related interactions, and hydrogen-bonding interactions, may contribute to compensating changes in electrostatic interactions. However, the present characterization does not allow the relative contribution of each mechanism to be quantified. Therefore, the results are more appropriately described as indicating a relatively broad pH tolerance of the adsorption system rather than complete pH independence.

Initial Concentration (Figure 6C): Increasing the concentration from 10 mg/L to 70 mg/L resulted in a significant increase in adsorption capacity (qe) (from 49.76 mg/g to 205.60 mg/g). This increase indicates that the driving force (concentration gradient) required for mass transfer at high concentrations is maximized. Operating in high concentration regions is advantageous for maximizing the mass transfer efficiency of the process, as the capacity tends to increase up to 70 mg/L. However, from a wastewater management perspective, the most economical operating range is the one where the adsorbent gives the highest qe value before reaching saturation, while simultaneously maintaining a residual concentration (Ce) that complies with discharge standards.

Contact Time (Figure 6D): Process kinetics show that adsorption progresses rapidly in the first 60 min and reaches equilibrium in the 120–150 min range. The qe value increased from 94.90 mg/g to 148.60 mg/g in the period from 10 min to 150 min. This indicates that the active sites are initially filled rapidly, and the system approaches its maximum adsorption efficiency with increasing time. The 120th minute, when the process reaches its highest efficiency, should be defined as the “optimum equilibrium time”. The capacity increase at the 150th minute is marginal (from 148.50 mg/g to 148.60 mg/g); therefore, terminating the process at 120 min saves energy and increases cost-effectiveness by extending the operating life of the orbital shaker.

### 2.3. Isotherm Model Fit Studies

**Figure 7:**
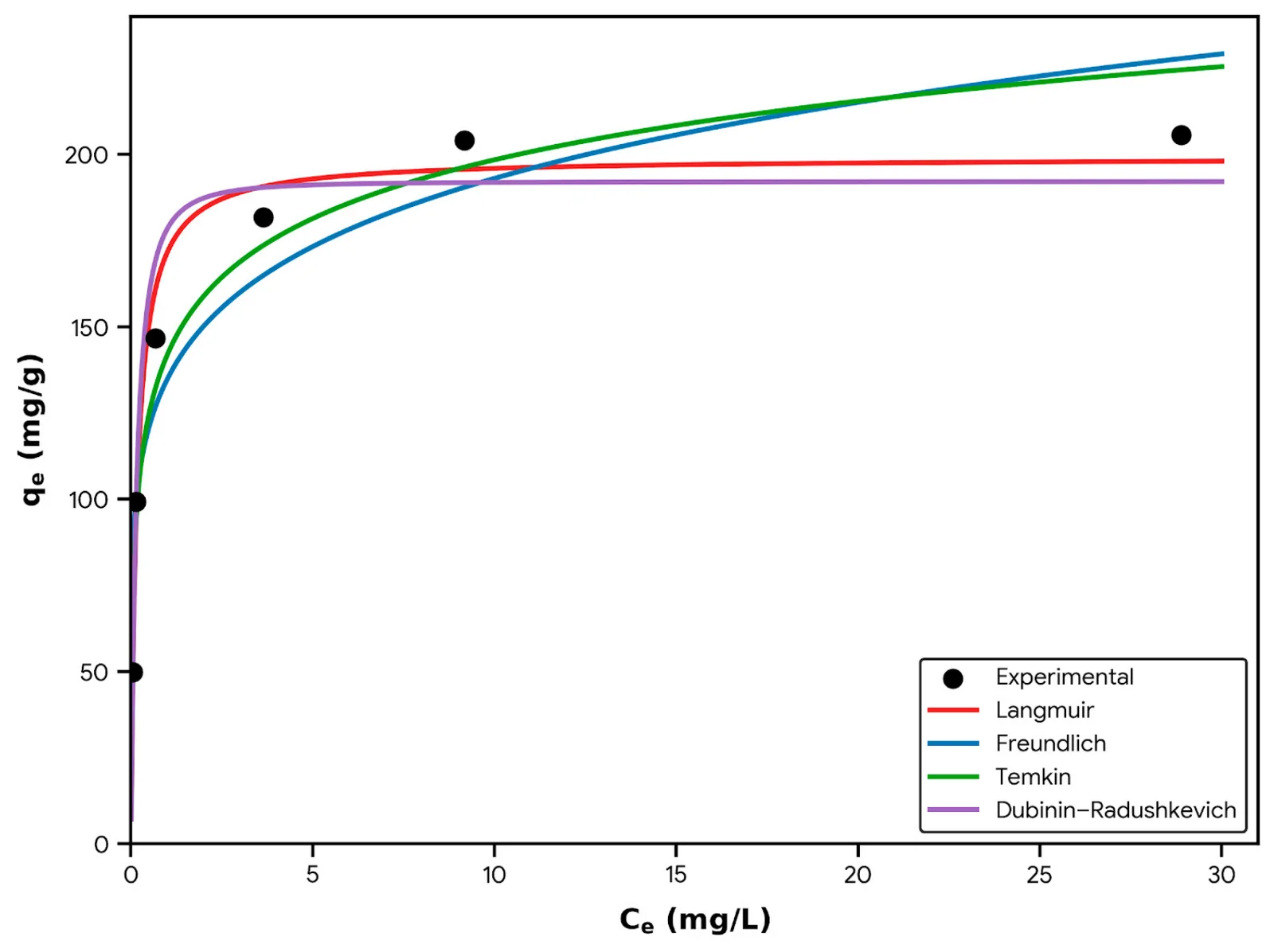
Isotherm model curves and experimental data points.

Isotherm data related to the adsorption process of activated carbon produced from hazelnut husks in Remdesivir removal were analyzed using non-linear regression methods. SSE, RMSE, and χ^2^ error analyses were performed to determine the fit between experimental data and theoretical models, and the results are presented below. All isotherm model parameters and error statistics calculated by non-linear regression from equilibrium data obtained at different initial concentrations are given in Table 1, and isotherm model curves and experimental data points are given in Figure 7.

Langmuir Model: The Langmuir model provided the best statistical description among the tested isotherm models, with R^2^ = 0.9759, R^2^adj = 0.9699, SSE = 472.59, RMSE = 8.875 mg g^−1^, and χ^2^ = 3.116. The calculated Langmuir capacity was qm = 199.11 mg g^−1^, while the experimentally measured maximum uptake was 205.60 mg g^−1^. The dimensionless separation factor RL ranged from 0.0158 to 0.0023 over the investigated initial concentration range, indicating favorable equilibrium behavior according to the mathematical interpretation of the Langmuir model.

The relatively good fit of the Langmuir equation indicates that its mathematical form describes the equilibrium data more effectively than the other models tested. However, the Langmuir equation is an empirical equilibrium model, and its statistical fit alone does not establish molecularly homogeneous adsorption sites or prove monolayer adsorption at the molecular level. The present result is therefore interpreted as evidence that the Langmuir formulation provides an appropriate description of the measured equilibrium behavior under the experimental conditions.

Temkin Model: The Temkin model, which assumes that the heat of adsorption decreases linearly as surface coverage increases due to interactions between adsorbent and adsorbate molecules, provided an acceptably good fit to the experimental data (R^2^ = 0.9462, R^2^_adj_ = 0.9328). Although the error metrics (SSE = 1056.08, RMSE = 13.267 mg/g, χ^2^ = 9.026) indicate higher deviations compared to the Langmuir model, it reflects the surface behavior more stable than the Freundlich model. The equilibrium binding constant A_T_ = 318.64 L/mg and adsorption energy constant b_T_ = 100.76 J/mol (B = 24.60 mg/g) calculated from the model reveal that the active centers on the surface of hazelnut husk activated carbon exhibit a homogeneous energy distribution and that adsorbent-solute interactions occur with moderate-low temperature changes.

The Dubinin–Radushkevich model yielded an average characteristic adsorption energy of E = 22.14 kJ mol^−1^. This parameter provides information related to the energetic characteristics of the adsorption process within the assumptions of the D–R model. However, the characteristic energy should not be interpreted independently as definitive evidence of possible chemical contribution because its physical interpretation depends on the model assumptions and the characteristics of the adsorbent–adsorbate system. Accordingly, the obtained E value is considered a supporting indication of relatively strong adsorbent–adsorbate interactions, rather than conclusive evidence of chemical bonding.

Freundlich Model: The Freundlich model, which is based on multilayer adhesion on heterogeneous surfaces, was the isotherm with the weakest fit to experimental data among the tested models (R^2^ = 0.8703, R^2^_adj_ = 0.8378). The highest deviation values in error analysis were observed in this model (SSE = 2547.34, RMSE = 20.605 mg/g, χ^2^ = 22.076); the model’s overestimation of capacity, particularly in the low equilibrium concentration region (C_e_ = 0.049 mg/L), constituted a significant portion of the total χ^2^ error. However, the fact that the model’s heterogeneity factor remained at n = 6.405 (1/n = 0.156 < 1) supports that the affinity between the adsorbent and Remdesivir molecules is highly favorable, even though the surface heterogeneity assumption remains weak.

These results reveal that hazelnut husk activated carbon has a very high and saturable adsorption capacity for Remdesivir, and the process is largely described by the Langmuir isotherm.

### 2.4. Kinetic Model Fit Studies

**Figure 8:**
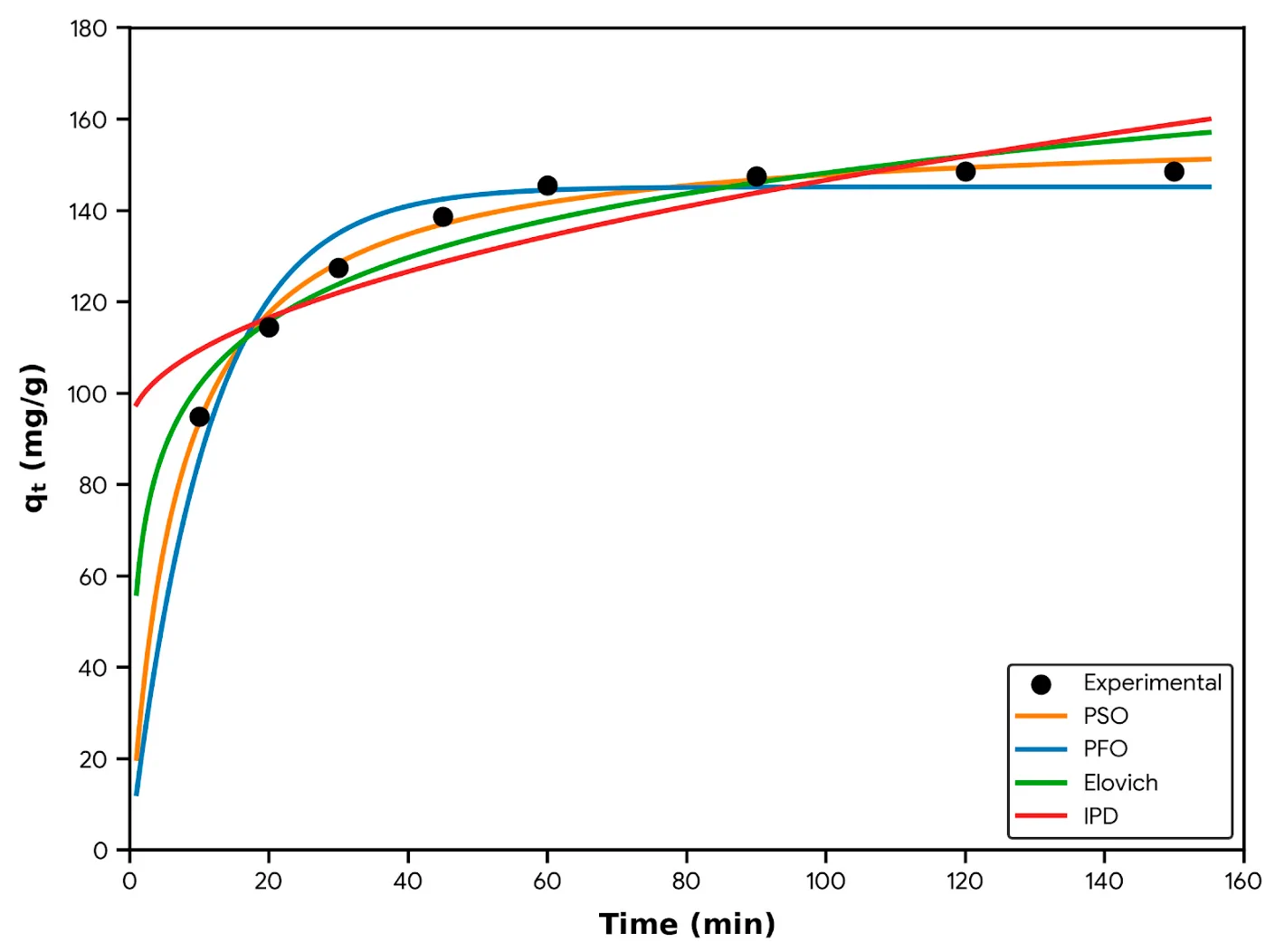
Kinetic model curves and experimental data points.

All kinetic model parameters and error statistics calculated by nonlinear regression from the experimental data are given in Table 2, and the corresponding model curves and experimental data points are presented in Figure 8.

Among the kinetic models evaluated, the pseudo-second-order (PSO) model provided the best statistical description of the experimental time-dependent adsorption data, with (R^2^ = 0.999), SSE = 1.82, RMSE = 0.58 mg g^−1^, and (χ^2^ = 0.02). The pseudo-first-order (PFO), Elovich, and intraparticle-diffusion (IPD) models showed comparatively larger deviations from the experimental data.

The superior statistical fit of the PSO model indicates that the mathematical form of this model describes the observed adsorption kinetics effectively. However, the PSO model is empirical, and a high correlation coefficient or low error values do not, by themselves, establish a possible chemical contribution mechanism or demonstrate the formation of chemical bonds.

The observed kinetic behavior may reflect the combined influence of surface-site accessibility, external mass transfer, pore diffusion, adsorbent–adsorbate interactions, and other transport or interfacial processes. Therefore, the PSO result is interpreted here as an empirical kinetic description of the experimental data rather than as independent evidence of possible chemical contribution.

The agreement between the equilibrium capacity estimated by the PSO model (151.15 mg g^−1^) and the experimentally observed value (148.60 mg g^−1^) further indicates that the PSO equation provides a close mathematical representation of the observed kinetic profile. However, this agreement is interpreted as evidence of model adequacy rather than evidence of a specific molecular adsorption mechanism.

These analyses demonstrate that activated carbon produced from hazelnut husks exhibits excellent performance in Remdesivir removal through favorable surface interactions, and that the PSO model is the kinetic model that most accurately describes the process.

### 2.5. Thermodynamic Investigation of Remdesivir Adsorption

Thermodynamic parameters were evaluated at 298, 303, 308, and 313 K using the temperature-dependent Langmuir affinity constants obtained from nonlinear equilibrium modeling. In the present study, the dimensional Langmuir affinity constant (K_L_) (L mg^−1^) was distinguished from the dimensionless standard-state equilibrium constant used for thermodynamic calculations.

The dimensionless equilibrium constant was obtained from the Langmuir affinity constant according to:

K_d_^0^ = K_L_ C^0^ (1)

where (C^0^) is the standard concentration of 1 mol L^−1^, expressed in the same concentration units as required for the cancellation of the units of (K_L_). Because the molecular weight of Remdesivir is 602.59 g mol^−1^, 1 mol L^−1^ corresponds to 602.59 mg L^−1^. Thus:

K_d_^0^ = K_L_ 602.59

The standard Gibbs free-energy change was calculated from:

ΔG^0^ = −RT ln K_d_^0^ (2)

where R = 8.314 J/(mol K) and T is the absolute temperature.

The standard enthalpy and entropy changes were determined from the Van’t Hoff relationship:

(3) ln Kd0=ΔS0R−ΔH0RT

Accordingly, linear regression of (ln Kd^0^) against (1/T) was performed using the four experimental temperatures (298, 303, 308, and 313 K). The slope corresponds to (−ΔH^0^/R), whereas the intercept corresponds to (ΔS^0^/R). The regression yielded ΔH^0^ = −49.29 kJ mol^−1^, ΔS^0^ = −4.77 J mol^−1^ K^−1^, and (R^2^ = 0.9965).

The negative value of (ΔH^0^) indicates (Table 3) that the overall adsorption process is exothermic over the investigated temperature range. The negative values of (ΔG^0^) indicate that the adsorption process is thermodynamically favorable under the investigated conditions. The negative (ΔS^0^) suggests a decrease in the overall entropy of the adsorption system upon transfer of the adsorbing species from the aqueous phase to the adsorbent surface.

Importantly, the thermodynamic equilibrium constant (K_d_^0^) used in this analysis is distinct from the dimensional distribution coefficient (q_e_/C_e_). The latter is not used directly in the Gibbs free-energy or Van’t Hoff calculations because it does not provide the same dimensionless standard-state equilibrium constant without an explicit standard-state conversion.

## 3. Discussion

In this study, the Remdesivir adsorption performance of activated carbon produced from hazelnut husk by KOH activation was investigated, and the findings were discussed by comparing them with the literature.

### 3.1. Adsorbent Characterization and Structure-Performance Relationship

The characterization results indicate that KOH activation produced a porous carbon material with a BET surface area of 441.72 ± 7.72 m^2^ g^−1^ and measurable contributions from both microporous and larger-pore domains. The t-plot external surface area of 299.54 m^2^ g^−1^ represents a substantial external-surface contribution; however, this parameter should not be directly equated with geometrically accessible mesopore surface area. The coexistence of microporosity and larger pores may nevertheless be relevant to the uptake of relatively large pharmaceutical molecules because pore accessibility can influence mass-transfer pathways.

The SEM observations showed an irregular and porous surface morphology, consistent with structural changes occurring during carbonization and chemical activation. EDX analysis indicated that carbon and oxygen were the dominant detected elements. The oxygen signal suggests the presence of oxygen-containing elemental species, but specific functional groups cannot be assigned from EDX alone. Consequently, possible contributions from oxygen-containing surface functionalities to adsorption are discussed only as plausible interactions rather than experimentally established molecular assignments.

The pHpzc of 6.466 indicates that the predominant surface charge state is expected to change around this pH. The relatively limited variation in adsorption capacity across the investigated pH range suggests that electrostatic interactions were not the only factor controlling adsorption. Pore accessibility and non-electrostatic interactions may also contribute to the observed behavior. Nevertheless, because FTIR/XPS characterization and direct molecular-level interaction measurements were not performed, the relative contribution of individual interaction mechanisms cannot be quantitatively established.

### 3.2. Effects of Adsorption Parameters

Adsorbent Dose: Increasing the activated carbon dose from 5 to 25 mg per 50 mL resulted in a substantial decrease in the equilibrium Remdesivir concentration, from 3.807 to 0.211 mg L^−1^ and consequently increased the removal efficiency from 87.31% to 99.30%. This behavior can be attributed to the increase in the number of available adsorption sites and total surface area with increasing adsorbent mass.

In contrast, the adsorption capacity (q_e_) decreased from 261.93 mg g^−1^ at 5 mg adsorbent to 59.58 mg g^−1^ at 25 mg adsorbent. The decrease in (q_e_) with increasing adsorbent dose is expected because the amount of Remdesivir available in the fixed solution volume is distributed over a progressively larger mass of adsorbent. Consequently, although the total removal from the aqueous phase increases, the amount adsorbed per unit mass of activated carbon decreases.

The corrected results therefore demonstrate two different effects of increasing adsorbent dose: increasing the dose improves the overall removal efficiency, whereas it reduces the adsorption capacity expressed per gram of adsorbent. At 10 mg/50 mL, a removal efficiency of 96.14% was achieved. Increasing the adsorbent dose from 10 to 15 mg increased the removal efficiency by only 0.95 percentage points, while further increases to 20 and 25 mg resulted in removal efficiencies of 98.59% and 99.30%, respectively. Thus, the additional removal achieved at higher doses was relatively limited compared with the corresponding increase in adsorbent consumption.

Accordingly, 10 mg/50 mL was selected as the practical optimum adsorbent dose for subsequent experiments because it provided a high Remdesivir removal efficiency while limiting the amount of activated carbon required. This selection was based on the balance between removal efficiency and adsorbent consumption rather than on the maximum (q_e_) value.

Solution pH: The experimentally determined adsorption capacity remained within a relatively narrow range of 148.32–145.72 mg g^−1^ over the investigated pH range of 2–12. This relatively limited variation indicates that the adsorption performance was not strongly affected by pH under the experimental conditions employed in the present study. Although Remdesivir-containing species may undergo changes in protonation state with pH, the relatively stable adsorption capacity suggests that electrostatic interactions alone may not control the overall adsorption behavior. Other contributions, including pore accessibility and non-electrostatic adsorbent–adsorbate interactions, may also participate.

However, this observation was obtained using controlled aqueous solutions and should not be directly extrapolated to real wastewater. Real wastewater contains competing dissolved species, natural organic matter, inorganic ions, and other constituents that may alter adsorbate speciation, surface interactions, and adsorption performance. Therefore, the relatively limited pH dependence observed here should be regarded as a laboratory-scale finding under the investigated conditions rather than as evidence that pH adjustment would be unnecessary in practical wastewater treatment [15].

Initial Concentration: Increasing the q_e_ value from 49.76 mg/g to 205.60 mg/g with increasing the concentration from 10 mg/L to 70 mg/L supports the decisive effect of the concentration gradient on mass transfer [25]. The capacity reached at 70 mg/L is quite close to the Langmuir theoretical maximum (199.11 mg/g).

Contact Time: Adsorption proceeded rapidly during the initial stage of the experiment and approached an apparent equilibrium within approximately 120 min under the investigated batch conditions. The adsorption capacity reached at 90 min was 146.80 mg g^−1^, compared with 148.50 mg g^−1^ at 120 min, corresponding to a difference of approximately 1.1%. This small difference indicates that the adsorption process had approached its apparent equilibrium level by approximately 90 min under the experimental conditions used. However, this observation should be interpreted only as a laboratory-scale kinetic finding. The 90-min contact time should not be considered a validated practical treatment time because process-scale performance depends on factors such as adsorbent loading, hydrodynamics, mass transfer, competing solutes, wastewater composition, and reactor configuration. Further continuous-flow and application-relevant studies are required to establish an appropriate contact time for practical treatment systems.

### 3.3. Kinetic Modeling of Experimental Data

The PSO model provided the best statistical fit to the experimental kinetic data, with (R^2^ = 0.999), SSE = 1.82, RMSE = 0.58 mg g^−1^, and (χ^2^ = 0.02). These results indicate that the mathematical form of the PSO model describes the observed time-dependent adsorption behavior more effectively than the PFO, Elovich, and intraparticle-diffusion models.

The close agreement between the PSO-derived equilibrium capacity (151.15 mg g^−1^) and the experimentally determined equilibrium capacity (148.60 mg g^−1^) further supports the suitability of the PSO equation for representing the observed kinetic profile. However, this statistical agreement should not be interpreted as proof of possible chemical contribution, chemical-bond formation, or a chemically controlled rate-limiting step.

The observed adsorption kinetics are likely influenced by more than one process. Surface-site accessibility, external mass transfer, pore diffusion, and adsorbent–adsorbate interactions may contribute simultaneously to the overall uptake rate. The intraparticle-diffusion model yielded (R^2^ = 0.924) and a non-zero intercept (C = 95.1), suggesting that intraparticle diffusion alone does not describe the entire kinetic process. This observation is consistent with the possibility that external mass transfer and pore transport contribute to the overall adsorption kinetics.

The Elovich model showed an intermediate statistical performance (R^2^ = 0.962), indicating that its mathematical form also captures part of the observed kinetic behavior. However, the Elovich fit is not interpreted as independent evidence of possible chemical contribution. Overall, the kinetic results indicate that the PSO equation provides the most appropriate empirical description among the tested models, while the experimental data do not permit assignment of a unique molecular adsorption mechanism solely from kinetic modeling.

### 3.4. Thermodynamic Evaluation

The negative standard enthalpy changes ΔH^0^ = −49.29 kJ/mol indicates the exothermic nature of Remdesivir adsorption onto hazelnut husk activated carbon under the investigated conditions. The negative standard entropy change ΔS^0^ = −4.77 J/(mol K) suggests a decrease in the overall configurational freedom of the adsorbed species and the adsorption system upon uptake. The negative (ΔG^0^) values obtained over the investigated temperature range indicate that the adsorption process is thermodynamically favorable under the experimental conditions.

The Dubinin–Radushkevich characteristic energy (E = 22.14 kJ/mol) provides an additional energetic descriptor of the adsorption process; however, this parameter is not considered sufficient by itself to establish chemical-bond formation or to distinguish unequivocally between possible chemical contribution and physisorption in a heterogeneous porous carbon system. Similarly, the favorable PSO fit is interpreted as an empirical description of the adsorption kinetics rather than as independent evidence of possible chemical contribution. Taken together, the thermodynamic and kinetic results describe the overall energetic and temporal behavior of the adsorption process but do not establish a unique molecular adsorption mechanism.

### 3.5. Proposed Adsorption Mechanism

Based on the textural characterization, pHpzc measurements, equilibrium behavior, kinetic results, and thermodynamic analysis, the adsorption of the Remdesivir-containing formulation onto hazelnut husk-derived activated carbon can be conceptually interpreted as the combined outcome of pore accessibility, mass-transfer processes, and possible non-covalent adsorbent–adsorbate interactions. The developed porous structure, characterized by the coexistence of microporous and larger-pore domains, may facilitate the transport of the adsorbed species toward accessible adsorption regions.

The relatively stable adsorption capacity over the investigated pH range suggests that electrostatic interactions alone may not control the overall adsorption behavior. Depending on the protonation state of the adsorbed species and the surface charge of the activated carbon, electrostatic attraction or repulsion may contribute to the adsorption process under specific conditions. In addition, π-related interactions and hydrogen-bonding interactions may represent possible non-covalent contributions to adsorption because of the carbonaceous surface and the chemical functionalities potentially present in the material. However, these interactions should be regarded as proposed or plausible contributions rather than experimentally established molecular interactions.

The kinetic results indicate that the adsorption process involves more than a single transport phenomenon. The good statistical performance of the pseudo-second-order model provides an empirical description of the observed time-dependent adsorption behavior, whereas the intraparticle-diffusion analysis suggests that pore diffusion may contribute to the overall process without necessarily being the sole controlling step. These results therefore support a multistep interpretation involving external mass transfer, pore transport, and adsorption at accessible surface regions, rather than a unique molecular rate-controlling mechanism.

The EDX analysis confirmed the presence of carbon and oxygen as major elemental components of the activated carbon; however, EDX does not provide the chemical-state information required to identify specific functional groups. Because FTIR, XPS, or other surface-sensitive spectroscopic analyses were not performed, the presence and contribution of specific oxygen-containing functional groups, as well as specific hydrogen-bonding or π–π interactions, cannot be established directly. Accordingly, the mechanistic interpretation presented here is considered a conceptual framework supported by the available physicochemical and adsorption data rather than a definitive molecular-level mechanism.

Overall, the experimental findings are consistent with a possible combination of pore accessibility, mass transfer, and non-covalent adsorbent–adsorbate interactions. Nevertheless, further surface-sensitive characterization, such as FTIR and XPS before and after adsorption, together with experiments using isolated Remdesivir and appropriate formulation/excipient controls, would be required to distinguish and quantify the individual molecular interactions involved.

### 3.6. Comparative Evaluation with the Literature

The experimentally determined maximum adsorption uptake in the present study was 205.60 mg g^−1^, while the nonlinear Langmuir model yielded a calculated capacity of 199.11 mg g^−1^. These values can be compared with previously reported adsorption capacities for biomass-derived activated carbons; however, direct comparison should be made cautiously because adsorption capacity depends strongly on the pollutant identity, initial concentration, adsorbent dose, solution pH, contact time, temperature, formulation composition, and equilibrium conditions.

The literature comparison presented in Table 4 is therefore intended to provide contextual information rather than establish a direct performance ranking among adsorbents. In particular, adsorption capacity and percentage removal are different performance parameters and should not be considered interchangeable. Differences in experimental conditions may substantially affect the reported qmax values.

The present material exhibited a BET surface area of 441.72 m^2^ g^−1^ and measurable contributions from microporous and larger-pore regions. These textural properties may be relevant to the adsorption of relatively large pharmaceutical molecules. Nevertheless, the present batch experiments were performed using a remdesivir-containing commercial formulation rather than an isolated analytical standard and did not involve real wastewater. Consequently, the results should be interpreted as an initial laboratory-scale assessment rather than direct evidence of treatment performance in real wastewater.

### 3.7. Limitations and Future Perspectives

Several limitations should be considered when interpreting the present findings. First, the adsorption experiments were conducted using a commercial remdesivir formulation containing SBECD rather than an isolated analytical Remdesivir standard. Consequently, the contribution of the excipient to adsorption, pore occupation, or chromatographic response cannot be completely separated from that of the active pharmaceutical ingredient. An SBECD-only placebo experiment was not performed in the present study.

Second, the initial concentrations investigated in the equilibrium experiments were selected for laboratory-scale adsorption modelling and do not represent the concentration range expected in typical environmental waters. Therefore, the reported adsorption capacities should not be interpreted as direct evidence of performance at environmentally relevant trace concentrations.

Third, the analytical method was based on HPLC-UV detection. Although the analyte was quantified according to its chromatographic retention behavior, independent peak-purity or LC–MS confirmation was not performed. Additional analytical validation would strengthen the specificity assessment in the presence of formulation excipients.

Fourth, the available characterization consisted primarily of BET, SEM, EDX, and pHpzc measurements. Because FTIR, XPS, or other surface-sensitive spectroscopic analyses were not performed, the assignment of specific oxygen-containing functional groups and molecular-level adsorption interactions remains tentative.

Finally, the present study was performed in batch aqueous systems and did not evaluate real pharmaceutical wastewater, multicomponent competition, adsorbent regeneration, long-term stability, or continuous-flow operation. Future studies should therefore investigate real wastewater matrices, trace-level concentrations, SBECD/placebo controls, complementary spectroscopic characterization, regeneration and reuse, and continuous-flow column performance.

## 4. Materials and Methods

### 4.1. Raw Materials and Pre-Treatment

Hazelnut husks, used as starting material in activated carbon production, were obtained from a hazelnut orchard in Zonguldak province, Turkey. The raw biomass was washed several times with deionized water to remove adhered dirt and surface impurities, then rinsed with deionized water to remove remaining soluble contaminants. The cleaned hazelnut husks were air-dried at room temperature until the moisture content dropped below 10%. Finally, to completely remove any remaining moisture, the material was dried in a laboratory-type oven at 105 °C for one day. The dried hazelnut husks were ground in a laboratory-type grinder (MM 400, Retsch GmbH, Haan, Germany). Subsequently, they were sieved using a test sieve (Retsch GmbH, Haan, Germany) to obtain homogeneous particles in the size range of 100–200 micrometers. This specific size range was chosen to ensure that the material mixes homogeneously with the chemical and activates in the same way each time.

### 4.2. Chemical Activation Process

Chemical activation was performed using potassium hydroxide (KOH). KOH is known as an effective activator in the production of high surface area and microporous activated carbon. In the activation process, KOH was mixed with biomass in a 1:1 mass ratio. First, KOH was dissolved in deionized water and mixed with the prepared hazelnut husk powder. To ensure homogeneous distribution of the activator in the biomass, the mixture was stirred continuously for 2 h and then left to stand at room temperature for 24 h. The chemically treated material was then dried at 105 °C until it reached a constant weight.

The activation process with KOH proceeds as a series of complex reduction-oxidation (redox) reactions under high temperatures. These reactions result in the formation of metallic potassium (K), potassium carbonate (K_2_CO_3_), hydrogen gas (H_2_), and carbon monoxide (CO). All these reactions contribute to the formation of a porous structure within the carbon skeleton. The metallic potassium released during activation penetrates the carbon structure, leading to expansion of the lattice structure and the formation of more pores. Furthermore, the formation of gaseous compounds facilitates the creation of micro and meso-sized pores. After the washing stage, the potassium-containing compounds are removed, leaving behind a highly porous activated carbon structure.

### 4.3. Carbonization

The dried and chemically treated samples were carbonized in a tube furnace (Protherm Furnaces, Ankara, Turkey) under an N_2_ flow rate of 1 L min^−1^. The furnace temperature was raised from room temperature to 850 °C at a heating rate of 10 °C min^−1^ and held isothermally at this temperature for 1 h. Following carbonization, the furnace was allowed to cool to 50 °C under continuous nitrogen flow. Based on preliminary screening experiments and relevant literature on lignocellulosic precursors [21,24], an impregnation ratio of 1:1 and an activation temperature of 850 °C were selected to maximize the balance between carbon yield and pore network development.

### 4.4. Washing and Finishing

The resulting activated carbon was subjected to a washing process to remove remaining inorganic substances and activation chemicals. First, the product was washed repeatedly with tap water. Then, it was treated with a 0.1 M HCl solution to remove potassium-based residues such as K_2_CO_3_ and other inorganic byproducts formed during activation. Afterwards, the material was rinsed with pure water until the pH reached the range of 6.5–7.5 to ensure that acid-soluble components were completely removed. In the final stage, the sample was rinsed with ethanol to facilitate moisture removal. The washed activated carbon was first dried by standing it at room temperature for 2 h, then dried in an oven at 105 °C until a constant mass was reached. The final product was stored in tightly sealed polyethylene containers prior to characterization and adsorption studies. The elemental composition was determined using energy-dispersive X-ray spectroscopy (EDX) coupled with SEM. To ensure representative and statistically robust compositional data, the spectra were collected via a multi-region area-scan mode across broad surface windows of multiple activated carbon particles rather than isolated single-point spots.

### 4.5. Remdesivir

**Figure 9:**
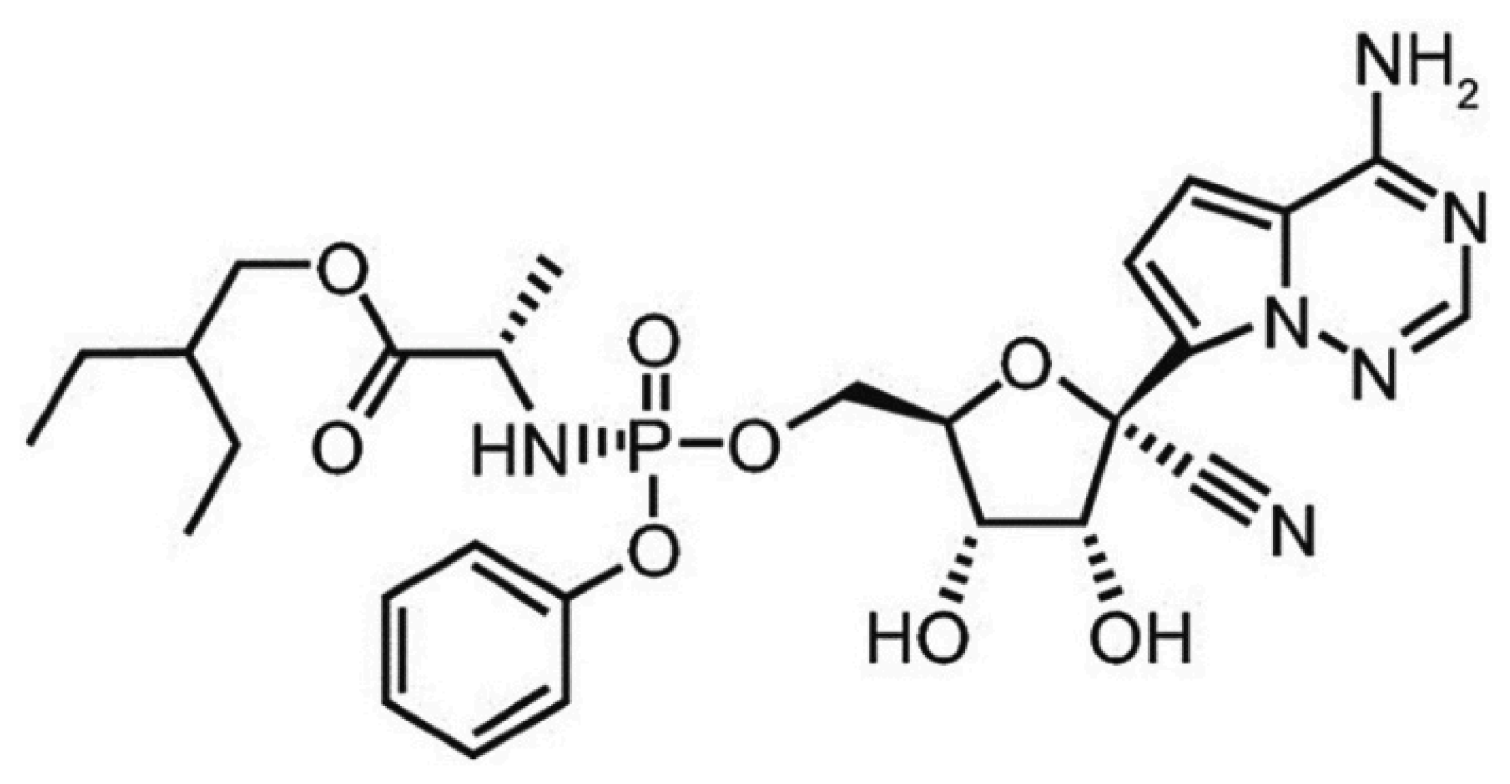
Molecular structure of remdesivir.

Remdesivir is a prodrug that is an analogue of adenosine triphosphate and exhibits antiviral activity. Its chemical formula is C_27_H_35_N_6_O_8_P, its molecular weight is 602.6 g/mol, and its maximum absorption wavelength is 246–247 nm. Remdesivir is produced as a single stereoisomer with six chiral centers and is a crystalline solid compound that is slightly soluble in water (Figure 9), has a log *p* value of 3.2, and a pKa of 3.3. After entering the cell, it inhibits viral polymerases and causes RNA chain termination. Remdesivir has shown in vitro efficacy against various RNA viruses, including MERS-CoV, SARS-CoV, and SARS-CoV-2, while in vivo studies have shown that it reduces lung viral titers and prevents weight loss in mouse models.

To prepare the stock Remdesivir (Covifor^®^, Hetero Labs Limited, Hyderabad, India) solution, 50 mg of Remdesivir was precisely weighed into a 100 mL beaker and dissolved in 20 mL of methanol. The solution was transferred to a 100 mL volumetric flask. The beaker was rinsed three times with deionized water, and the contents were transferred to the volumetric flask. The volumetric flask was stirred for 10 min, and the volume was completed with deionized water to obtain a concentration of 500 µg/mL. The final solution was filtered through Whatman No. 42 filter paper (pore size 2.5 µm). Remdesivir is a prodrug that is an analogue of adenosine triphosphate and exhibits antiviral activity. Its chemical formula is C_27_H_35_N_6_O_8_P, its molecular weight is 602.6 g/mol, and its maximum absorption wavelength is 246–247 nm. Remdesivir is produced as a single stereoisomer with six chiral centers and is a crystalline solid compound that is slightly soluble in pure water (Figure 9), with a reported log *p* value of ~3.2 and a pKa of ~3.3.

To overcome the inherent low aqueous solubility of pure remdesivir and to mimic pharmaceutical-grade discharge matrices, a commercial formulation (Covifor^®^, lyophilized powder) containing sulfobutylether-β-cyclodextrin sodium (SBECD) as a solubilizing and stabilizing excipient was utilized. The presence of SBECD forms stable host-guest inclusion complexes that significantly enhance remdesivir’s apparent aqueous solubility by orders of magnitude compared to the free-base form.

To prepare the stock remdesivir solution (500 mg/L), an accurately weighed amount of the formulation equivalent to 50 mg of active remdesivir was dissolved initially in 20 mL of HPLC-grade methanol to ensure complete molecular dispersion. The dissolved mixture was transferred quantitatively to a 100 mL volumetric flask, the dissolution container was rinsed three times with deionized water, and the flask was adjusted to volume with deionized water. The resulting stock solution was stirred for 15 min and filtered through a 0.22 μm PTFE membrane filter to confirm total clarity, absence of particulates, and complete dissolution. Working solutions were subsequently prepared by volumetric dilution with deionized water.

### 4.6. Analytical Method

Chromatographic analysis was performed using a high-performance liquid chromatography (Agilent Technologies, Waldbronn, Germany) equipped with a UV–Vis detector. The analysis was conducted using an Agilent Extend C18 column (4.6 mm × 250 mm, 5.0 µm) at 25 °C. The mobile phase consisted of 20 mM potassium dihydrogen phosphate solution and acetonitrile at a 50:50 (*v*/*v*) ratio and was filtered through a 0.22 µm membrane before use. The flow rate was 1.2 mL min^−1^, the detection wavelength was 247 nm, and the total run time was 10 min. The retention time assigned to Remdesivir was approximately 3.86 min.

Because the experiments employed a commercial formulation containing SBECD and other formulation components, chromatographic selectivity is an important consideration. The present study identified the analyte according to its chromatographic retention behavior under the stated HPLC-UV conditions; however, independent peak-purity analysis or LC–MS confirmation was not performed. Similarly, an SBECD-only placebo control was not analyzed. Therefore, the possibility of minor matrix-related contributions or co-elution cannot be completely excluded and is acknowledged as a limitation of the analytical procedure. Future work should include placebo controls and/or orthogonal analytical confirmation, such as LC–MS or validated peak-purity assessment, particularly when analyzing complex pharmaceutical formulations.

### 4.7. Batch Adsorption Experiments

Batch adsorption experiments were conducted to evaluate the performance of activated carbon produced from hazelnut husk in the adsorption of remdesivir from aqueous solutions. In this context, five key parameters affecting adsorption efficiency were systematically investigated: adsorbent dose, solution pH, initial remdesivir concentration, contact time, and temperature.

The adsorption efficiency of adsorbent dose was investigated using a univariate methodology. While all other parameters (initial concentration, temperature, stirring speed, contact time) were kept constant, only the amount of adsorbent was changed. The experiments were performed in 250 mL Erlenmeyer flasks with caps, using 50 mL of remdesivir solution (30 mg/L). Different amounts (5, 10, 15, 20, and 25 mg/50 mL) of activated carbon from hazelnut husk were added to each flask. The study was conducted under standardized conditions (25 ± 2 °C, 180 rpm) for 2 h (120 min). At the end of this period, the contents of the bottles were filtered through white band filter paper, and the remdesivir concentration in the filtrate was analyzed using an HPLC device. The optimum adsorbent dose was determined by calculating the adsorption capacity (q, mg/g) and removal efficiency (%).

To determine the effect of different initial concentrations on adsorption, working solutions ranging from 20 to 100 mg/L (10, 20, 30, 40, 50, 70 mg/L) were prepared by diluting a 500 mg/L stock remdesivir solution at appropriate ratios. All experiments were conducted in 250 mL Erlenmeyer flasks with caps, using 50 mL of remdesivir solution. 10 mg of activated carbon from hazelnut husk was added to each flask at the optimum adsorbent dose. The study was conducted under standardized conditions (25 ± 2 °C, 180 rpm) for 120 min. At the end of the period, the contents of the flasks were filtered, and the remdesivir concentration in the filtrate was determined. To determine the effect of temperature on adsorption, experiments were conducted at four different temperatures (298, 303, 308, and 313 K). Experiments were performed in 250 mL Erlenmeyer flasks with caps, using 50 mL of remdesivir solution (30 mg/L). 10 mg of activated carbon from hazelnut husk was added to each flask. The mixture at 298 K was shaken for 120 min in a temperature-controlled shaker operating at 180 rpm. The mixture at 313 K was shaken in a hot bath for 120 min in a temperature-controlled shaker operating at 180 rpm. At the end of the period, the contents of the flasks were filtered through Whatman No. 42 filter paper (Whatman International Ltd., Maidstone, UK), and the remdesivir concentration in the filtrate was determined. Working solutions covering the range of 10 to 70 mg/L were prepared by diluting the 500 mg/L stock solution with deionized water. In all working solutions, remdesivir remained fully solubilized, transparent, and stable without phase separation or supersaturation effects, aided by the trace cosolvent presence and the inclusion complexation provided by the SBECD excipient present in the Covifor^®^ matrix. Control test flasks containing remdesivir solutions without adsorbent were agitated in parallel under identical conditions; no decrease in remdesivir concentration was observed, confirming the absence of precipitation or non-specific vessel-wall sorption during the batch tests.

When investigating the pH effect, the pH of the solution was adjusted to values of 2, 4, 7, 10, and 12 using 0.1 M HCl or 0.1 M NaOH solutions. Following pH adjustment, 10 mg of activated carbon was added to each flask in 50 mL of remdesivir solution, and the mixtures were shaken at 180 rpm for 120 min.

The contact time range (0–180 min) was chosen based on equilibrium times observed in preliminary experiments to allow for complete monitoring of adsorption kinetics and reliable determination of equilibrium time. All experiments were conducted in 250 mL Erlenmeyer flasks containing 50 mL of remdesivir solution (30 mg/L) and 10 mg of activated carbon (produced from hazelnut husks) as adsorbent. The flasks were shaken at 180 rpm in a temperature-controlled orbital shaker maintained at 25 ± 2 °C. Samples taken at the specified time intervals (10, 20, 30, 60, 90, 120, 150, and 180 min) were filtered, and the remdesivir concentration in the filtrate was determined. After the contact time, the solution was filtered, and the remaining concentrations were analyzed by HPLC. The adsorption capacity was calculated using the following equation [31]:

(4) $$q_{e}=\frac{\left(C_{0}-C_{e}\right)V}{m}$$

Here, (*C*_0_) and (*C_e_*) are the initial and equilibrium concentrations (mg/L), respectively, (V) is the solution volume (L), and (m) is the amount of biosorbent (g).

### 4.8. Isotherm Modeling

Equilibrium adsorption data were analyzed using the nonlinear forms of the Langmuir, Freundlich, Temkin, and Dubinin–Radushkevich (D–R) models (Table 5). The Langmuir equation provides a saturation-type equilibrium description commonly associated with an idealized homogeneous-site model, whereas the Freundlich equation describes heterogeneous equilibrium behavior. The Temkin model accounts empirically for changes in adsorption energy with surface coverage, while the D–R model provides an energetic descriptor of the adsorption process. In the present study, these equations were used as mathematical models to quantitatively describe the experimental equilibrium data. The assumptions associated with each model were not considered direct experimental evidence of molecular surface homogeneity, complete monolayer coverage, or a specific adsorption mechanism. Nonlinear regression analysis was performed to determine the model parameters. Goodness of fit was evaluated using statistical error functions, including root mean square error (RMSE), sum of squared errors (SSE), and chi-square test (χ^2^).

### 4.9. Kinetic Modeling

Kinetic experiments were performed at room temperature (25 ± 2 °C). In 50 mL of solution with an initial remdesivir concentration of 30 mg/L, 10 mg (0.2 g/L) of activated carbon (adsorbent) obtained from hazelnut husks was used. Samples were shaken at 180 rpm in a temperature-controlled shaker. Samples were taken at the specified time intervals (10, 20, 30, 60, 90, 120, 150, 180 min), filtered through a 0.22 μm syringe filter, and the residual remdesivir concentration was determined by HPLC-UV. The amount adsorbed at time t, q_t (mg/g), was calculated using Equation (1). The obtained experimental data were fitted with pseudo-first-order (PFO), pseudo-second-order (PSO), Elovich, and intraparticle diffusion (IPD, Weber-Morris) models using non-linear regression. The best model was determined based on the R^2^ value, error analyses (SSE, RMSE, χ^2^), and residual distribution. Non-linear forms of pseudo-first-order (PFO), pseudo-second-order (PSO), Elovich, and intraparticle diffusion (IPD) models were used to describe adsorption kinetics (Table 6).

### 4.10. Thermodynamic Analysis of Adsorption

Batch adsorption experiments were conducted at 298, 303, 308, and 313 K to evaluate the thermodynamic characteristics of the adsorption process. At each temperature, 50 mL of Remdesivir solution with an initial concentration of 30 mg L^−1^ was contacted with 10 mg of hazelnut husk-derived activated carbon for 120 min at 180 rpm under temperature-controlled conditions. Following equilibration, the solutions were filtered and the residual Remdesivir concentrations were determined by HPLC.

The Langmuir affinity constant (K_L_), obtained from nonlinear Langmuir equilibrium modeling at each temperature, was used as the starting equilibrium parameter for the thermodynamic analysis. (K_L_) has units of L mg^−1^ and was not used directly in the thermodynamic equations [40,41,42].

To obtain a dimensionless standard-state equilibrium constant, (K_L_) was converted according to:

K_d_^0^= K_L_ C^0^

where (C^0^) is the standard concentration of 1 mol L^−1^. For Remdesivir (MW = 602.585) g mol^−1^:

C^0^ = 602.59 g/L = 602.59 mg/L

Therefore:

K_d_^0^= K_L_ 602,585

When (K_L_) is expressed in L mg^−1^. The resulting (K_d_^0^) is dimensionless.

For example, at 298 K, (K_L_ = 406.31) L mg^−1^ gives: 2.44836 × 10^8^.

The standard Gibbs free-energy change was calculated from:

ΔG^0^ = −RT ln K_d_^0^

where (R = 8.314) J mol^−1^ K^−1^ and (T) is the absolute temperature.

The standard enthalpy and entropy changes were determined from the Van’t Hoff equation:

ln Kd0=ΔS0R−ΔH0RT

Thus, the slope of the plot of (ln Kd0) versus (1/T) is (−ΔH^0^/R), while the intercept is (ΔS^0^/R).

Four temperature points (298, 303, 308, and 313 K) were included in the regression.

The dimensional distribution coefficient (qe/Ce) was not used as the thermodynamic equilibrium constant because its numerical value and units depend on the concentration and capacity units used and it is not identical to the dimensionless standard-state equilibrium constant (K_d_^0^).

All thermodynamic calculations were performed using the unrounded (K_L_) values generated by the nonlinear Langmuir regression. Rounded values are presented in Table 3 for reporting purposes. The final thermodynamic parameters were obtained from the regression based on the unrounded dataset.

### 4.11. Error Analysis

The statistical parameters in Table 7 were used to evaluate model fit.

### 4.12. Data Modeling and Statistical Analysis

To quantitatively evaluate the equilibrium and kinetic adsorption behavior of the Remdesivir-containing formulation onto hazelnut husk-derived activated carbon, experimental data were analyzed using nonlinear regression approaches. Kinetic data were fitted to the pseudo-first-order (PFO), pseudo-second-order (PSO), Elovich, and intraparticle-diffusion (IPD/Weber–Morris) models. These models were used to compare their ability to describe the observed time-dependent adsorption behavior and to assess the possible contribution of transport and diffusion processes.

The PSO model was evaluated as an empirical kinetic equation describing the relationship between adsorption capacity and contact time. Its statistical performance was compared with the other kinetic models using goodness-of-fit and error criteria. A high PSO fit was therefore interpreted as evidence that the mathematical form of the model adequately represents the experimental kinetic profile under the investigated conditions. It was not interpreted as direct evidence of possible chemical contribution, chemical bond formation, or a specific surface reaction.

The IPD model was used to assess whether intraparticle diffusion could contribute to the overall adsorption kinetics. Deviation of the IPD relationship from the origin was considered in conjunction with the other kinetic results and was interpreted as indicating that pore diffusion may not be the only transport contribution. However, the kinetic models were not used to assign a unique molecular-level rate-controlling mechanism.

Equilibrium adsorption data were analyzed using the nonlinear forms of the Langmuir, Freundlich, Temkin, and Dubinin–Radushkevich (D–R) models. These models were used as mathematical descriptors of the equilibrium relationship between adsorbate concentration and adsorption capacity. The Langmuir model was used to evaluate the suitability of a saturation-type equilibrium relationship, the Freundlich model to describe heterogeneous equilibrium behavior, the Temkin model to evaluate the concentration dependence of the adsorption energy, and the D–R model to obtain an energetic descriptor of the adsorption process. The parameters obtained from these models were interpreted as model-dependent descriptors and were not considered, individually or collectively, to constitute direct molecular evidence for a specific adsorption mechanism.

Model performance was evaluated using the coefficient of determination (R^2^), the agreement between experimental and calculated adsorption capacities, and statistical error functions including sum of squared errors (SSE), root mean square error (RMSE), and chi-square (χ^2^). The model showing the best statistical agreement was considered the most appropriate empirical descriptor among the tested models under the experimental conditions. Statistical model fitting was therefore used to quantitatively describe the observed adsorption behavior rather than to establish a unique molecular mechanism.

All data analyses, nonlinear regression calculations, curve fitting, and statistical evaluations were performed using OriginPro 2021 software. Experimental measurements were evaluated using at least three parallel replicates, and the results were reported as mean ± standard deviation.

**Figure 10:**
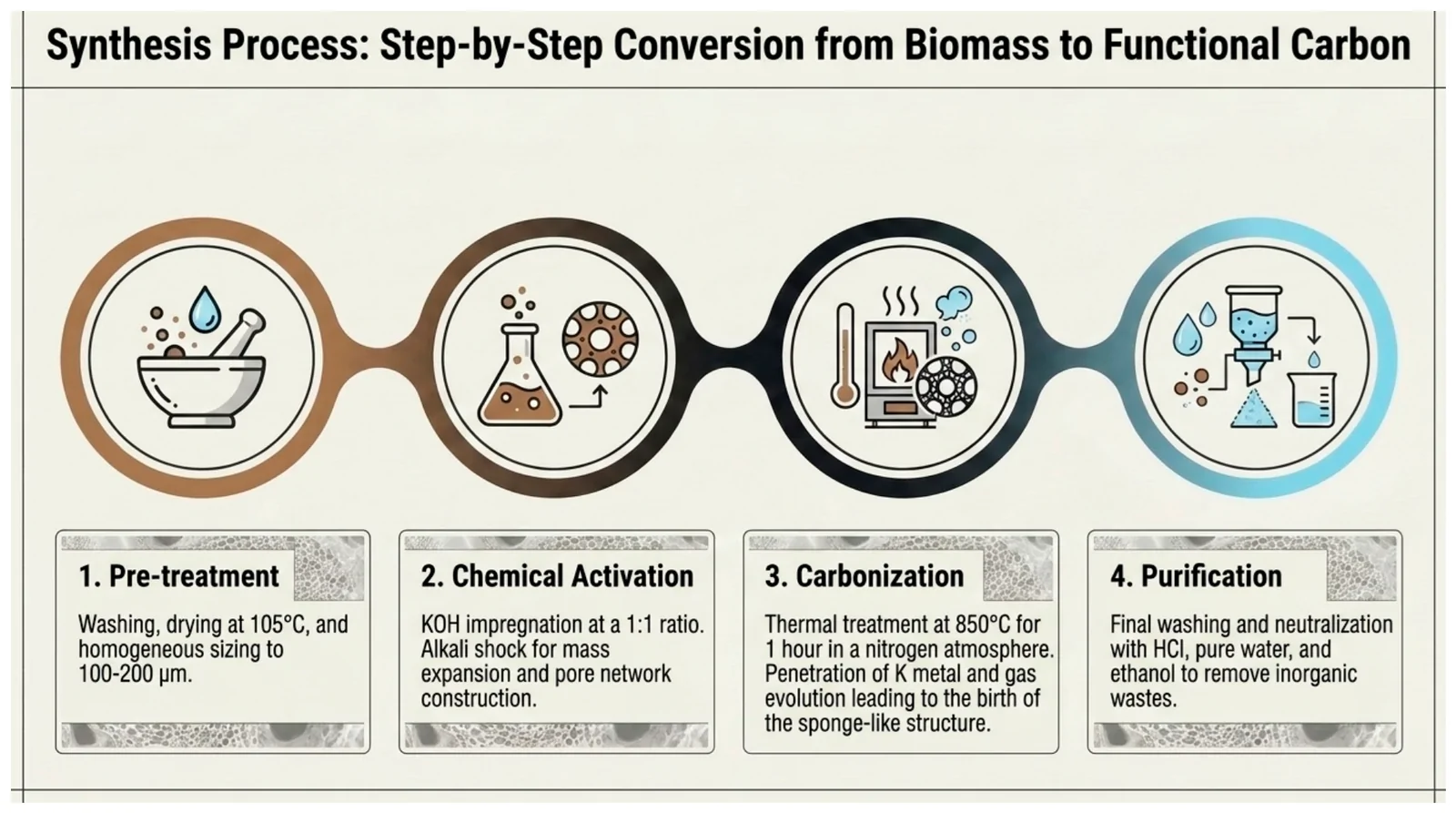
Schematic illustration of the step-by-step synthesis process for converting hazelnut husk biomass into functional activated carbon: (1) Pre-treatment: Washing, drying at 105 °C, and homogeneous sizing to 100–200 µm; (2) Chemical Activation: KOH impregnation at a 1:1 ratio for mass expansion and pore network development; (3) Carbonization: Thermal treatment at 850 °C for 1 h under an N_2_ atmosphere; and (4) Purification: Final washing and neutralization using HCl, pure water, and ethanol to remove inorganic impurities.

The overall material preparation pathway, adsorption-related processes, and potential application of hazelnut husk-derived activated carbon are conceptually summarized in Figure 10. The schematic illustrates how the porous structure may facilitate adsorbate transport and accessibility to internal adsorption regions. It also depicts possible non-covalent interactions, including π-related and hydrogen-bonding interactions, as potential contributions to the overall adsorption behavior. These interactions are shown as proposed possibilities rather than experimentally established molecular mechanisms. The final part of the schematic represents the potential relevance of the material for aqueous pharmaceutical contaminant removal; however, direct applicability to municipal or hospital wastewater requires further validation using real wastewater matrices, formulation/excipient controls, regeneration studies, and continuous-flow experiments.

## 5. Conclusions

In this study, activated carbon prepared from hazelnut husk waste through KOH-assisted chemical activation was evaluated for the adsorption of a remdesivir-containing commercial pharmaceutical formulation from aqueous media. The activated carbon exhibited a BET surface area of 441.72 ± 7.72 m^2^ g^−1^ and a porous structure containing both microporous and larger-pore contributions.

The equilibrium data were described most effectively by the nonlinear Langmuir model among the tested isotherm equations, yielding a calculated adsorption capacity of 199.11 mg g^−1^ (R^2^ = 0.9759), while the experimentally measured maximum uptake was 205.60 mg g^−1^. The Langmuir result is interpreted as an empirical description of the equilibrium behavior and is not taken as direct proof of molecularly homogeneous adsorption sites or complete monolayer coverage.

The adsorption kinetics were best described by the pseudo-second-order model (R^2^ = 0.999). This result indicates that the PSO equation adequately represents the observed time-dependent adsorption behavior under the investigated conditions; however, it does not, by itself, establish possible chemical contribution, chemical bond formation, or a specific molecular rate-controlling mechanism. The intraparticle-diffusion analysis further suggests that pore diffusion may contribute to the overall process but is not the only transport contribution.

Thermodynamic analysis yielded a negative enthalpy change (ΔH° = −49.29 kJ mol^−1^) and negative Gibbs free-energy values over the investigated temperature range, indicating a favorable and exothermic adsorption process under the experimental conditions. The combined textural, pHpzc, equilibrium, kinetic, and thermodynamic results are consistent with a possible contribution of pore accessibility, mass transfer, and non-covalent adsorbent–adsorbate interactions. However, the available characterization does not establish a definitive molecular adsorption mechanism, and specific interactions such as hydrogen bonding or π-related interactions remain proposed rather than directly demonstrated.

Therefore, the mechanistic scheme presented in this study should be regarded as a conceptual interpretation of the available experimental evidence rather than a definitive molecular model. Further investigations using FTIR, XPS, appropriate formulation/excipient controls, real wastewater matrices, regeneration experiments, and continuous-flow systems are required to identify the individual molecular contributions and to assess practical treatment applicability.

An important limitation is that the experiments were conducted under controlled batch conditions using a commercial formulation containing SBECD rather than isolated Remdesivir in a real wastewater matrix. Accordingly, the present results represent an initial laboratory-scale assessment of the adsorption behavior and should not be interpreted as direct evidence of treatment performance in real wastewater. Further studies involving SBECD/placebo controls, competing solutes, real wastewater matrices, trace-level concentrations, complementary surface-sensitive characterization, regeneration, continuous-flow operation, and process-relevant hydraulic conditions are required before practical treatment applicability can be assessed.

## Figures and Tables

**Table 1:** Isotherm model parameters and statistical analysis table.

Isotherm Model	Model Parameters	Value	Error Analysis Parameters
Langmuir	q_m_ (mg/g) K_L_ (L/mg) R_L_ (C_0_ = 10 → 70 mg/L	199.11 6.230 0.0158 → 0.0023	R^2^ = 0.9759 R^2^_adj_ = 0.9699 SSE = 472.59 RMSE = 8.875 mg/g χ^2^ = 3.116
Temkin	A_T_ (L/mg) B (mg/g) b_T_ (J/mol)	318.64 24.60 100.76	R^2^ = 0.9462 R^2^_adj_ = 0.9328 SSE = 1056.08 RMSE = 13.267 mg/g χ^2^ = 9.026
Dubinin–Radushkevich (In molar terms: mol/L and mol/g)	q_D_ (mg/g) β (mol^2^/kJ^2^) E (kJ/mol)	418.77 1.020 × 10^−3^ 22.14	R^2^ = 0.9078 R^2^_adj_ = 0.8848 SSE = 4.984 × 10^−9^ (mol/g)^2^ RMSE = 2.882 × 10^−5^ mol/g χ^2^ = 2.714 × 10^−5^ mol/g
Freundlich	K_F_ [mg/g)(L/mg)^1/n^] n 1/n	134.76 6.405 0.156	R^2^ = 0.8703 R^2^_adj_ = 0.8378 SSE = 2547.34 RMSE = 20.605 mg/g χ^2^ = 22.076

**Table 2:** Kinetic model parameters and error analysis parameters.

Models	q_e_ (mg/g)	Model Parameters	R^2^	SSE	RMSE	χ^2^
PFO	148.82	k_1_: 0.0522 (1/min)	0.981	12.45	1.53	0.11
PSO	151.15	k_2_: 0.0005 (g/mg·min)	0.999	1.82	0.58	0.02
Elovich	**-**	α: 18.5; β: 0.05	0.962	24.90	2.16	0.18
IPD	**-**	k_id_: 4.25; C: 95.1	0.924	48.20	3.01	0.35

**Table 3:** Thermodynamic parameters for Remdesivir adsorption onto hazelnut husk activated carbon at different temperatures.

T (K)	K_L_ (L/mg)	Kd^0^ (10^8^)	ΔG^0^ (kJ/mol)	ΔH^0^ (kJ/mol)	ΔS^0^ (J/mol K)	R^2^
298	406.31	2.448	−47.86	−49.29	−4.77	0.9965
303	301.09	1.814	−47.91			
308	207.32	1.249	−47.74			
313	159.55	0.961	−47.83			

**Table 4:** Comparative evaluation of hazelnut husk-derived activated carbon with commercial carbons and biomass-based adsorbents for pharmaceutical and antiviral removal.

Adsorbent Matrix	Precursor/Type	Activation Route	SBET (m^2^/g)	Target Pollutant	qmax (mg/g)	Equilibrium Time/pH Range	Reference
Hazelnut husk AC	Agricultural residue	KOH (1:1, 850 °C)	441.7	Remdesivir	199.11	120 min/ pH 2–12	This study
Commercial Carbon (F400)	Bituminous coal	Steam physical	950–1050	Antiviral pharmaceuticals	110–145	>240 min/ pH 6–8	[15,29]
Commercial AC (Norit)	Peat/Coal	Chemical/Steam	850–1000	Antibiotics/Antivirals	125–160	180 min / pH dependent	[19,30]
Macadamia nutshell AC	Agro-industrial shell	H_3_PO_4_ chemical	580.4	Antiretroviral drugs	195.20	180 min/ pH 4–8	[15]
Date stone AC	Biomass seed	ZnCl_2_ chemical	612.0	Ceftriaxone	180.50	120 min/ pH 6–9	[19]
Walnut shell AC	Agro-waste shell	Steam/Physical	410.0	Ciprofloxacin/Antibiotics	150–250	150 min/ pH 5–7	[20]
Rice husk AC	Crop residue	NaOH chemical	320.5	Diclofenac	168.30	120 min/ pH 4–8	[22]

**Table 5:** Isotherm models used in modeling experimental data.

Models	Equation No	Non-Linear Forms	Reference
Langmuir Isotherm	(5)	$q_{e}=\frac{q_{\max}K_{L}C_{e}}{1+K_{L}C_{e}}$	[32]
Freundlich Isotherm	(6)	qe=KFCe1n	[33]
Temkin Isotherm	(7)	*q_e_* = *B* ln(*A C_e_*)	[34]
Dubinin–Radushkevich (D–R) Isotherm	(8)	*q_e_* = *q_m_* exp (−*K ε*^2^)	[35]

**Table 6:** Kinetic models used in modeling experimental data.

Kinetic Models	Equation No	Non-Linear Forms	Reference
PFO	(9)	$q_{t}=q_{e}(1-e^{-k_{1}t})$	[36]
PSO	(10)	$q_{t}=\frac{q_{e}^{2}k_{2}t}{1+q_{e}k_{2}t}$	[37]
Elovich Model	(11)	$q_{t}=\frac{1}{\beta }\ln(1+\mathrm{\alpha \beta t})$	[38]
IPD	(12)	*q_t_* = *k_id_t*^1/2^ + *C*	[39]

**Table 7:** Error analysis functions.

Statistical Error Functions	Equation No	Calculation Formulas
Sum of Squared Errors (SSE)	(13)	$\mathrm{SSE}=\sum {\left(q_{\exp}-q_{\mathrm{cal}}\right)}^{2}$
Root Mean Square Error (RMSE)	(14)	$\mathrm{RMSE}=\sqrt{\frac{1}{n}}\sum {\left(q_{\exp}-q_{\mathrm{cal}}\right)}^{2}$
Chi-square (χ^2^)	(15)	$\chi^{2\;}=\sum \frac{{\left(q_{\exp}-q_{\mathrm{cal}}\right)}^{2}}{q_{\mathrm{cal}}}$

## Data Availability

The original contributions presented in this study are included in the article. Further inquiries can be directed to the corresponding author.

## Abbreviations

The following abbreviations are used in this manuscript:

BET	Brunauer–Emmett–Teller
BJH	Barrett–Joyner–Halenda
D–R	Dubinin–Radushkevich
EDX	Energy-dispersive X-ray spectroscopy
HPLC	High-performance liquid chromatography
IPD	Intraparticle diffusion
